# An Overview of Dynamic Descriptions for Nanoscale Materials in Particulate Photocatalytic Systems from Spatiotemporal Perspectives

**DOI:** 10.1007/s40820-025-01687-3

**Published:** 2025-03-21

**Authors:** Jiawei Yan, Zhidong Wei, Kai Takagi, Masaya Motodate, Zhi Jiang, Chiaki Terashima, Wenfeng Shangguan

**Affiliations:** 1 Research Center for Combustion and Environment Technology, Shanghai Jiao Tong University, Shanghai, 200240 People’s Republic of China; 2 Research Institute for Science and Technology, Tokyo University of Science, Chiba, 278-8510 Japan; 3 College of Smart Energy, Shanghai Jiao Tong University, Shanghai, 200240 People’s Republic of China

**Keywords:** Dynamic description, Particulate photocatalytic system, Nanoscale photocatalyst, Spatiotemporal characterization

## Abstract

Particulate photocatalytic systems using nanoscale photocatalysts have been developed as an attractive promising route for solar energy utilization to achieve resource sustainability and environmental harmony. Dynamic obstacles are considered as the dominant inhibition for attaining satisfactory energy-conversion efficiency. The complexity in light absorption and carrier transfer behaviors has remained to be further clearly illuminated. It is challenging to trace the fast evolution of charge carriers involved in transfer migration and interfacial reactions within a micro–nano-single-particle photocatalyst, which requires spatiotemporal high resolution. In this review, comprehensive dynamic descriptions including irradiation field, carrier separation and transfer, and interfacial reaction processes have been elucidated and discussed. The corresponding mechanisms for revealing dynamic behaviors have been explained. In addition, numerical simulation and modeling methods have been illustrated for the description of the irradiation field. Experimental measurements and spatiotemporal characterizations have been clarified for the reflection of carrier behavior and probing detection of interfacial reactions. The representative applications have been introduced according to the reported advanced research works, and the relationships between mechanistic conclusions from variable spatiotemporal measurements and photocatalytic performance results in the specific photocatalytic reactions have been concluded. This review provides a collective perspective for the full understanding and thorough evaluation of the primary dynamic processes, which would be inspired for the improvement in designing solar-driven energy-conversion systems based on nanoscale particulate photocatalysts.

## Highlights

- The dynamic descriptions for nanoscale particulate photocatalysts have been elucidated in terms of the irradiation field, photo-excited carrier behavior and interfacial reaction in the photocatalytic systems.
- The advanced spatiotemporal characterization techniques and evaluation methods are collected with the introduction of recent works and applications.
- The challenges and prospects in the elaborate investigation of photocatalytic dynamics are discussed.

## Graphical abstract

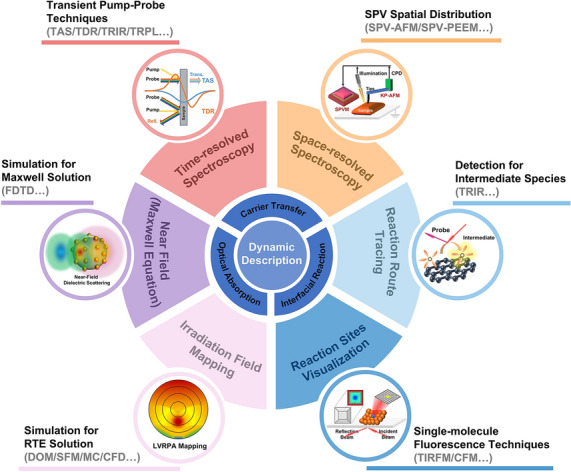

## Introduction

The past few decades have witnessed rapid development in photocatalytic systems, including applying a wide variety of nanoscale particulate photocatalytic materials, elucidating in-depth photocatalytic mechanisms, proposing multiple photo-induced redox reactions, and designing photocatalysis-based devices from bench scale to pilot test [1–6]. Despite remarkable breakthroughs in elevating apparent quantum efficiency and solar energy-conversion efficiency, there still exists a considerable gap in realizing the industrial application prospect, especially for overall water splitting and carbon dioxide reduction systems [7–9]. Dynamic obstacles in the whole photocatalytic process within a single-particle semiconductor micro-nanomaterial are considered as the main inhibition of achieving the target efficiency, which has gradually gained much more research concern in recent years. To distinguish the essential factor in photocatalytic dynamic behavior, the intrinsic correlation between energy flow and mass flow in the nanoscale and ultrafast timescale should be revealed. Charge carriers, participating in both two flows, are regarded as the substantial “shuttle” in the conversion of solar energy to chemical energy, and hence, kinetical analysis for charge carriers is supposed to be conducted. Based on three primary steps in the whole photocatalytic process: solar-light absorption, photo-induced charge carrier separation and transfer, and interfacial redox catalytic reactions, charge carrier’s behavior could be described correspondingly. In the irradiation, a quantity of non-equilibrium charge carriers is injected and empowered by incident photons (labeled as Step 1). Then, the transfer of photo-induced charge carrier occurs with the concomitant recombination (labeled as Step 2), in which the temporarily empowered solar energy is converted to heat by electron relaxation. After the directional migration, photo-induced charge carrier gets involved in the surface redox reaction (labeled as Step 3), and consequently, solar energy is stored in chemical energy. According to the previous research, a significant difference in the carrier lifetime (ranging from 10^–15^ to 10^–3^ s) could be ascribed to the mismatching of the reaction timescale among three steps [4, 9]. Obviously, the inconsistent carrier lifetime contributes to a higher probability of carrier recombination, thus leading to unsatisfactory efficiency. The prevailing thermodynamic strategies for photocatalytic materials including defect-related and junction engineering, and band structure alignment, etc., have been widely applied to prolong carrier lifetime and suppress carrier decay in recombination and trapping, which consequently improve the overall photocatalytic performance [2, 4, 10–15]. Therefore, the carrier lifetime in the decay process could be selected as the key parameter to describe temporal behaviors of photo-excited charge carriers in photocatalysis, which could be reflected by variable temporal characterization measurements. Complementary to the temporal perspective of separation and transfer behaviors of charge carriers, spatial behaviors rely on the spatial distribution of electrons and holes pairs. The adjustment of diffusion distance such as particle-size controlling and core–shell structure, and introduction of symmetric breaking, such as asymmetric co-catalyst assembling and anisotropic facet exposure, are bound to regulate the spatial distribution of charge carriers, which could be monitored by the spatial-resolved techniques and assisted with microscopy characterization. Combined with the parameter of carrier lifetime, the direct spatial imaging of carrier distribution that provides a concretization of separation and transfer process would be employed to evaluate the utilization efficiency of carrier kinetics and create inspiration for more precise and targeted designing concepts for more effective photocatalytic systems.

For the purpose of designing a high-efficiency photocatalytic system, improving the dynamic behavior of photo-induced charge carriers should be given a top priority via carrier regulation strategies. In addition, innovative characterization techniques have been widely applied for the reflection of dynamic photocatalytic processes more precisely in elaborate spatial scales and temporal scales. As mentioned above, three primary steps in photocatalytic systems (regarded as optical absorption step, carrier separation and transfer step and interfacial reaction step) are still encountered with respective dynamic obstacles and bottlenecks in each step, which require specific determination methods and characterization measurements to solve the targeted problem and study the principal contradiction in the key process. In this review, we summarize emerging and prevalent characterization techniques of spectroscopy and microscopy for the dynamic analysis of photo-induced charge carriers from the kinetic perspective. The dynamic behaviors of charge carriers, involving in the separation and migration within a single-particle photocatalyst material and surface photocatalytic reactions within the local interfacial region, have been illustrated by specific experimental measurements. In addition, we introduce various effective simulation methods with numerical modelings for optimal solar-light absorption and utilization in the irradiation field, which are applied to the fabrications of material designing and structural engineering. We comprehensively conclude the relationships between mechanistic conclusions from variable spatiotemporal characterization measurements and performance results of photocatalytic activity in the specific photocatalytic reactions. We also stress the significance of characterization findings for the corresponding photocatalytic performances and valuable guidance of characterization results for the novel designs of nanomaterial photocatalysts and the suitable selection of photocatalytic reactions and conditions in photocatalytic systems.

## Dynamics in Optical Absorption

In semiconductor theory, incident photons with sufficient energy ($hv > E_{g}$) are required to excite ground-state electrons from the valence band level up to the conduction band level ($E_{g}$ represents the band gap) in a single-particle semiconductor material. During the excitation, photo-induced electrons are endowed with photon energy and transited to the conduction band, while the inevitable dissipation of excess energy ($hv - E_{g}$) occurs via relaxation. Multiple exciton generation would be triggered particularly in nanoscale semiconductors, in which one high-energy photon ($hv > n \times E_{g}$) could induce multiple ($n$) electrons simultaneously [16]. Narrowing band gap of semiconductor photocatalyst, as a conventional strategy, has been applied to lift optical absorption ability with good response to wide wavelength range. The absorption ability is frequently measured by UV–Vis diffusion reflectance spectra (DRS) and the band gap is calculated by Tauc plot.

The improvement in the utilization of incident photon flux in the irradiation field deserves more attention, as it is estimated that almost half of the incident energy would be lost before the interaction with photocatalysts [9]. Generally, long-wavelength light tends to be absorbed by the multiphase medium, particularly in liquid-phase photocatalytic reactions. Besides, incident photon flux should decrease to an extent by scattering and transmission. Therefore, the regulation of the photon flux path is pivotal to the optimization of incident energy management. Although it is difficult for real-time tracing measurement, the dynamic behavior of photon flux could be analyzed via mathematic calculation methods [17, 18]. Previous reports have developed various simulation models with being verified in the corresponding photoreactor configuration, respectively. This review concludes two prevalent widely used models, the modified radiative transfer equation (RTE) in the irradiation field and the finite-difference time-domain model (FDTD) in the near-field for the dynamic description.

### Simulation for RTE Solution

Theoretical RTE is generally used to describe radiative transfer with energy transfer as electromagnetic radiation. Local volumetric rate of photo-absorption (LVRPA), which is regarded as one of the most significant physical parameters in the dynamic model of photocatalytic reactions, could be obtained through the RTE solutions as well as local volumetric rate of energy absorption (LVREA). The parameter LVRPA could be used as a measurement index to depict the irradiation field and analyze the optical absorption and utilization efficiencies, which supplies the evaluation of sufficient irradiation conditions created by the applied photoreactor configuration. The radiation dynamic transport could be explained as the combination of an increase from emission photons, a decrease from absorption photons, an increase from in-scattering photons and a decrease from out-scattering photons (Eq. 1) [19, 20]. In the equation, $I$ is specific irradiation intensity, $\kappa$ is absorption coefficient, $\sigma$ is scattering coefficient, $p$ is scattering phase junction, $s$ is spatial coordinate, and $j^{e}$ is emission intensity. Specifically, the spectral specific intensity $I_{\lambda , \Omega }$ depends on the direction propagation Ω and traveling penetration distance *s*.

1 $$ \frac{{\partial I_{\lambda , \Omega } \left( {s,t} \right)}}{\partial s} + \kappa_{\lambda } \left( {s,t} \right)I_{\lambda , \Omega } \left( {s,t} \right) + \sigma_{\lambda } \left( {s,t} \right)I_{\lambda , \Omega } \left( {s,t} \right) = j_{\lambda }^{e} \left( {s,t} \right) + \frac{{\sigma_{\lambda } \left( {s,t} \right)}}{4\pi }\mathop \smallint \limits_{{\Omega^{\prime}}}^{ } p\left( {\Omega^{\prime} \to \Omega } \right)I_{\lambda , \Omega } \left( {s,t} \right){\text{d}}\Omega^{\prime} $$

As the representative indicator in the description of incident irradiation field distribution, LVREA could be derived from the parameter $I_{\lambda , \Omega }$ with the absorption coefficiency $\kappa_{\lambda }$ according to Eq. 2. Systematically, the photon absorption rate for the photocatalytic system in the incident irradiation field could be obtained by following procedures: formulating RTE for the selected geometry; inputting appropriate absorption and scattering coefficiency, and phase function; accomplishing RTE solution [21].

2 $$ e_{\lambda }^{a} \left( {x,t} \right) = \mathop \smallint \limits_{\lambda }^{ } \kappa_{\lambda } \left( {x,t} \right){\text{d}}\lambda \mathop \smallint \limits_{\Omega }^{ } I_{\lambda , \Omega } \left( {x,t} \right){\text{d}}\Omega $$

3 $$ L\frac{{\partial I_{\lambda } \left( {s,t} \right)}}{\partial s} + \kappa_{\lambda } \left( {s,t} \right)I_{\lambda } \left( {s,t} \right) + \sigma_{\lambda } \left( {s,t} \right)I_{\lambda } \left( {s,t} \right) = \frac{{\sigma_{\lambda } \left( {s,t} \right)}}{2}\mathop \smallint \limits_{{L^{\prime}}}^{ } p\left( {L,L^{\prime}} \right)I_{\lambda } \left( {s,t} \right){\text{d}}L^{\prime} $$

**Fig. 1:**
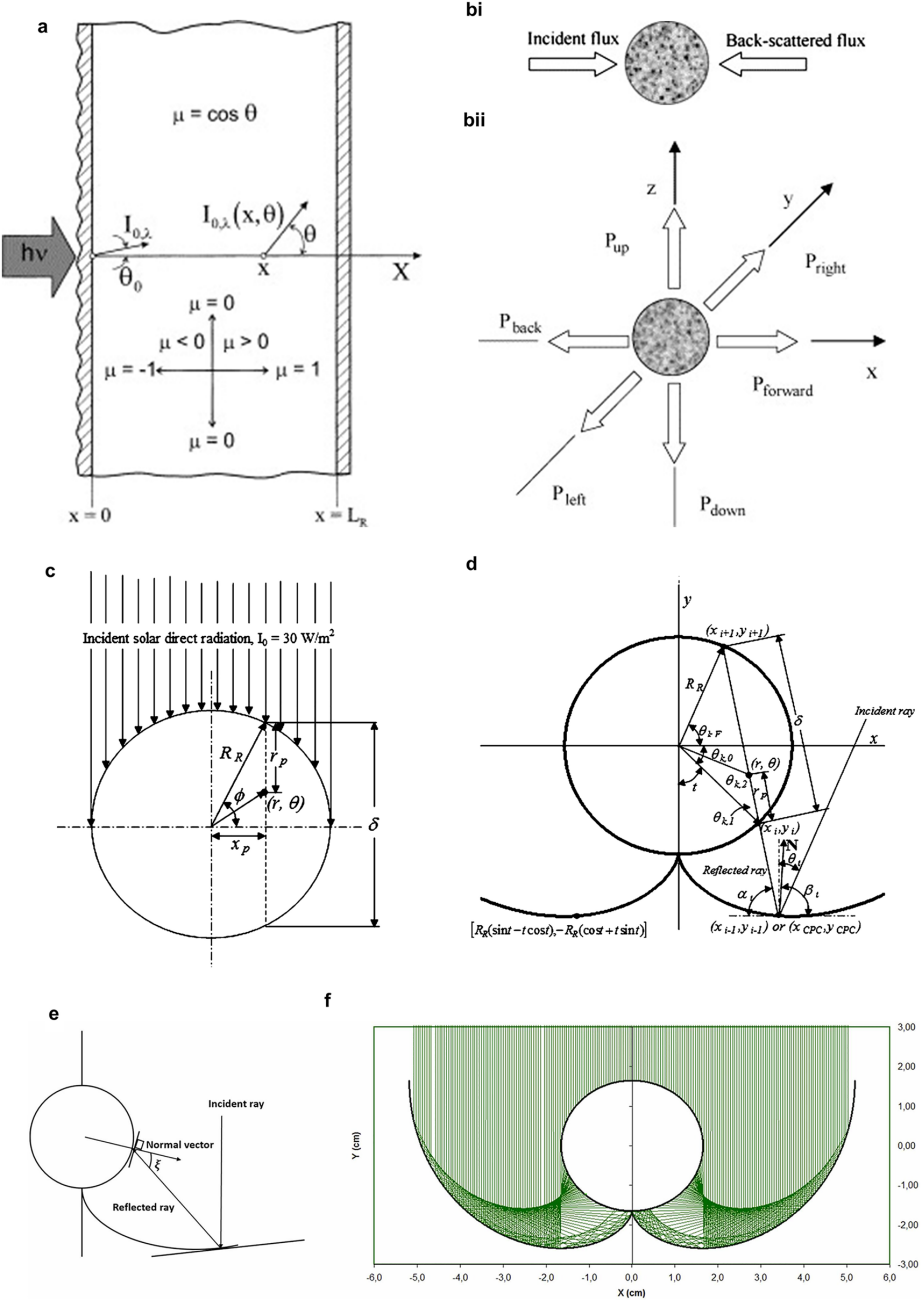
Common geometric configurations with variable simulation models. **a** One-dimensional simulation model. Reproduced with permission from Ref. [32]. Copyright © 2000 Elsevier. **bi** Two-flux model (TFM) and **bii** six-flux model (SFM). Reproduced with permission from Ref. [24]. Copyright © 2007 Elsevier. The travel directions of direct and reflected radiation light for **c** tubular and **d** CPC reactor configurations. Reproduced with permission from Ref. [26]. Copyright © 2010 American Chemical Society. **e** Ray tracing model and the corresponding **f** radiation simulation for CPC reactor configuration. Reproduced with permission from Ref. [19]. Copyright © 2021 Elsevier

However, RTE is an integro-differential equation without a simple analytical solution. Various modifications and adjustments toward solving RTE have been proposed, including discrete ordinate method (DOM) [22, 23], P1 approximation, two-flux and six-flux configurations [24–27], Monte Carlo strategy (MC) [28, 29] and computational fluid dynamics method (CFD) [30, 31]. DOM is regarded as a powerful numerical solution method in the generalized transport theory and hence could be applied to describe the photon transport model due to the similar mathematical features with that in neutron transport. Based on DOM, RTE could be transformed and enabled to be solved by the integration calculation. Without restrictions about geometry parameters and scattering anisotropy, DOM is universally applied for most types of photoreactors, including plate-flat type and cylindrical type. However, precise predictions via DOM adaptive for the above general conditions require significant computing time complexity and limited accuracy of boundary conditions (Eq. 3) [32], where $L$ is the direction cosine of propagating trajectory in terms of coordination $x$ (Fig. 1a).

Optimal and simplified calculation models have been proposed correspondingly for specific types of photoreactors. To simplify the radiation attenuation terms in RTE, a two-flux absorption–scattering model (TFM) (Fig. 1bi) is taken into consideration and has been further used to describe the irradiation field in terms of thin-film types with liquid flow along a flat-wall (FW) and along a internal wall (IW), coupling with characteristic emission models, respectively, Liner Source with Parallel Planes emission model (LSPP) and Linear Source with Spherical Emission (LSSE) [33]. Basically, the effective photon flux in LSPP is assumed to travel only along parallel planes orthogonal to the liquid film, whereas LSSE has the assumption of isotropic emitting along all directions. For both thin-film FW and IW types, the two-flux model states that the radiation at a random point is contributed by two photon fluxes, an incident flux in the forward direction and a fraction of backscattering in the backward direction. Furthermore, a novel six-flux absorption–scattering model (SFM) (Fig. 1bii) is developed as a three-dimensional extension of the above SFM with additional assumptions as follows [24, 26, 27]. Photons are either absorbed or scattered upon one photocatalyst particle, while scattering is allowed randomly in one of the six principle directions of the Cartesian coordinates (Fig. 1d). It is worth emphasizing that SFM in conjunction with ray tracing technique could give a sufficient description for general annular reactors (Fig. 1c) and compound parabolic collectors reactor (CPC) (Fig. 1e, f), with more simplified and accurate estimation of LVRPA (Eq. 4) [26].

4 $$ {\text{LVRPA}} = \frac{{I_{0} }}{{\lambda_{{\omega_{{{\text{corr}}}} }} \omega_{{{\text{corr}}}} \left( {1 - \gamma } \right)}}\left[ {\left( {\omega_{{{\text{corr}}}} - 1 + \sqrt {1 - \omega_{{{\text{corr}}}}^{2} } } \right)e^{{ - \frac{{r_{p} }}{{\lambda_{{\omega_{{{\text{corr}}}} }} }}}} + \gamma \left( {\omega_{{{\text{corr}}}} - 1 - \sqrt {1 - \omega_{{{\text{corr}}}}^{2} } } \right)e^{{\frac{{r_{p} }}{{\lambda_{{\omega_{{{\text{corr}}}} }} }}}} } \right] $$

where $I_{0}$ is the specific irradiation intensity of incident flux through the wall of the photocatalytic system, $\omega_{{{\text{corr}}}}$ is the corrected scattering albedo calculated from the optical property, $\lambda_{{\omega_{{{\text{corr}}}} }}$ is the extinction length, $\gamma$ is the apparent optical thickness, $r_{p}$ is the coordinate distance in the traveling incident direction.

**Fig. 2:**
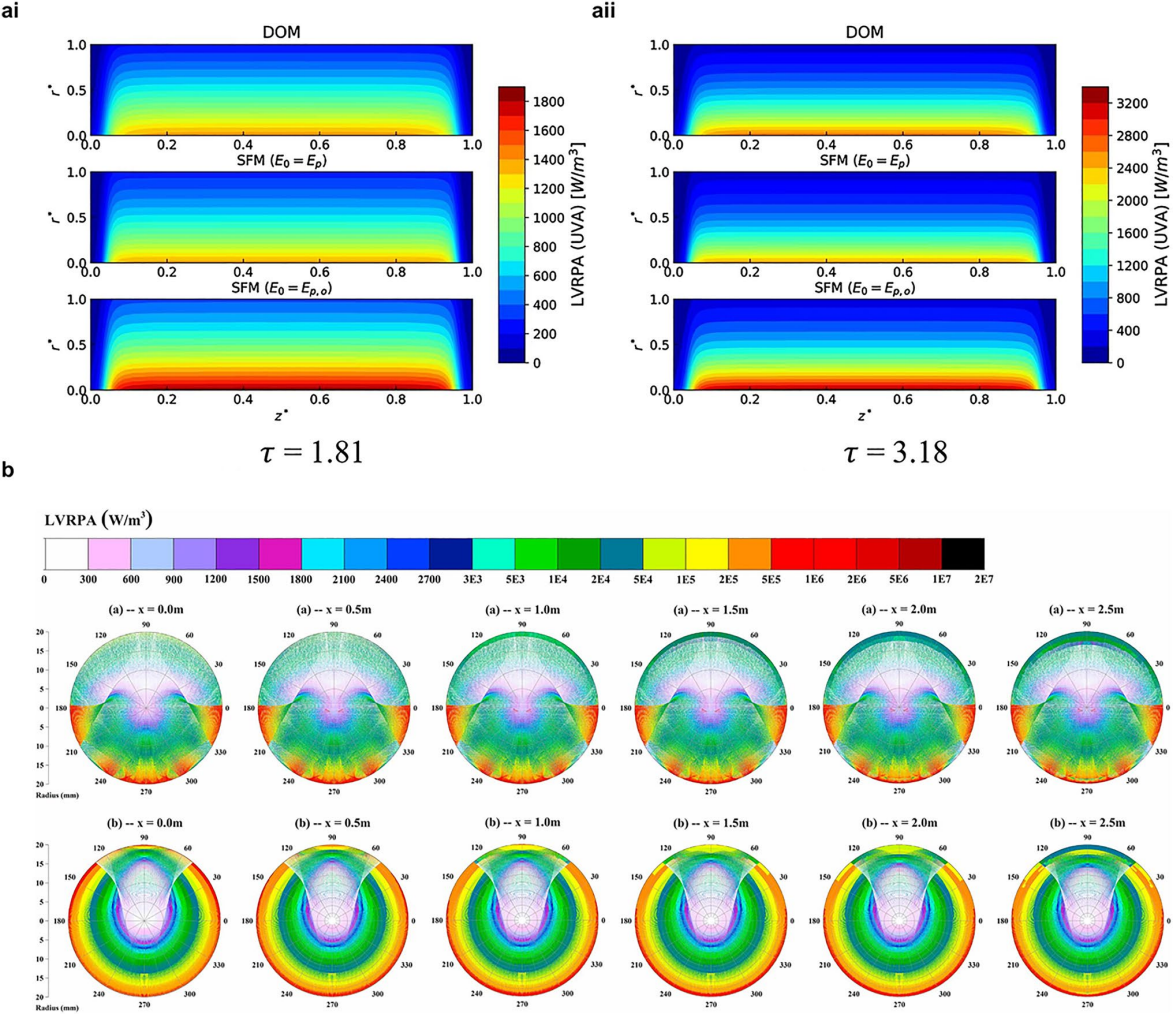
LVRPA distribution simulated by variable models for different configurations. The simulated LVRPA profiles obtained by DOM and SFM for annular reactor configurations with optical thickness **ai** *τ* = 1.81 and **aii** *τ* = 3.18. Reproduced with permission from Ref. [30]. Copyright © 2021 Elsevier. **b** Simulated LVRPA profiles obtained by SFM for CPC (top lane) and SUC (bottom lane) reactor configurations with the geometric concentration ratio (CR = 4.0). Reproduced with permission from Ref. [31]. Copyright © 2018 Elsevier

LVRPA distribution in different reactor configurations could be simulated by variable solution models with appropriate computed conditions (Fig. 2). In the contrast (Fig. 2ai, aii) between two solution methods (DOM and SFM) with boundary conditions (irradiance (${\text{SFM}}_{{\left( {E_{0} = E_{P} } \right)}}$) and fluence rate (${\text{SFM}}_{{\left( {E_{0} = E_{P,0} } \right)}}$)) and medium conditions (different optical thickness depending on particulate photocatalyst concentrations), SFM simulation with ${\text{SFM}}_{{\left( {E_{0} = E_{P} } \right)}}$ condition has a better approximation of LVRPA profile derived from DOM simulation than that with ${\text{SFM}}_{{\left( {E_{0} = E_{P,0} } \right)}}$ [30]. The more accurate evaluation of LVRPA distribution indicates SFM method using irradiance (${\text{SFM}}_{{\left( {E_{0} = E_{P} } \right)}}$) as boundary condition could be applied for annular photoreactors and other configuration with lateral symmetric geometry. It should be noted that CPC has been regarded as one of the most efficient solar configurations for pilot- and full-scale applications [26, 27, 31]. The decreasing tendency of LVRPA along with the distance to photoreactors’ center is found from the spatial cross-section profiles of LVRPA within CPC and SUC (surface uniform concentrator) configurations (Fig. 2b). The concentrated incident rays are observed near the scope of 0°, 180°, 240°, 270°, and 330° for CPC, while on the contrary, a more uniform radiation distribution ranging from 50° to 130° with a mild decreasing is obtained for SUC [31]. SFM also has been verified as a good agreement with the MC simulation result in planar reactors. For more complicated constructions requiring more rigorous models, MC simulation method is given high priority to deal with multi-parameters in geometry. As a powerful tool using random samplings to simulate the solution of physical and mathematical issues, MC solution could be applied to the present irradiation field by dissecting into several sequences [28]. The first event labeled as GENERATION describes the photon emission from the irradiation source and involves three random numbers in terms of the photon wavelength, the photon emission point and the photon direction of travel. The second event labeled as TRANSPORT evaluates the traveling length of photons. The last event labeled as INTERACTION involves all possibilities of photons being scattered, absorbed and transmitted. MC simulation tracks every individual photon from generation to ultimate photon-surface interaction in the traveling route [29, 34]. In specific, the solution of nonlinear equations for both geometry and photon trajectory, the calculation of the parametric distance between photon and surface and the stochastical determination of all photon-surface interaction fates should be taken into full consideration. For the cases that involve complex fluid and flow types, in addition to the combination of three transfers (heat, momentum, energy) and chemical reaction, CFD simulation has been widely applied with various developed numerical models [30, 31]. The efficient and accurate simulation methods and calculation models for the irradiation field help to design and verify optimal configurations of photoreactors and propose auxiliary equipment for solar energy concentration in the scaled continuous photocatalytic systems.

### Simulation for Maxwell Equation Solution

In addition to the intrinsic absorption within band-edge range derived from band structure, the extended absorption for longer wavelengths could be realized via the localized surface plasmon resonances (LSPRs) effect. LSPRs effect is introduced by plasmonic metallic nanostructures in conjunction with semiconductors and refers to the collective electron charge oscillations in plasmonic metals due to the strong resonant interaction with incident photons [35, 36]. The nanoscale near-field is highly localized at metallic nanostructures with much-enhanced amplitude at the resonance wavelength and consequently contributes to the generation of hot electrons with high-energy levels. Hence, the extension of absorption ability is attributed to the injection of energetic electrons from surface metallic nanostructures to the conduction band of semiconductors via metal plasmon energy transfer. The LSPRs intensity as well as the corresponding resonant photon wavelength could be modulated by the element, size and shape of metallic nanostructures and the surrounding environment.

**Fig. 3:**
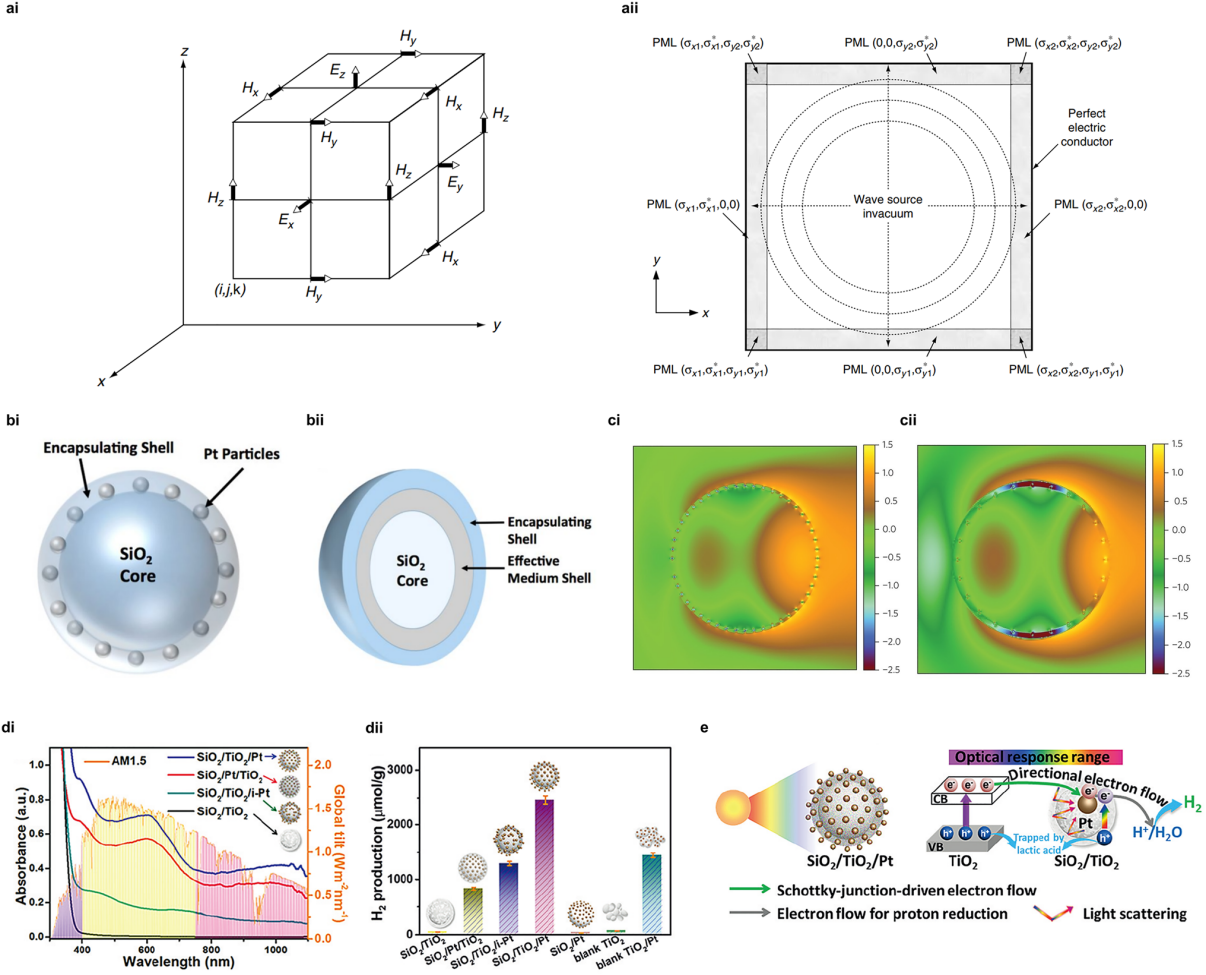
Illustration of FDTD solutions and unique absorption models designed for promoted optical absorption via near-field dielectric scattering. **ai** Positions of the electromagnetic-field vector components in the elementary cubic cell of the Yee space lattice with **aii** the computational domain terminated by the Berenger PML ABC. Reproduced with permission from Ref. [42]. Copyright © 2005 Elsevier. The theoretical models for SiO_2_ support are modified by positively charged APTES and negatively charged Pt nanoparticles, denoted as **bi** Pt/SiO_2_-SA and further coated by TiO_2_ shell, denoted as **bii** Pt/SiO_2_@TiO_2_ structures. **ci–cii** Electric field distributions $In\left( {\left| {E_{x} } \right|^{2} } \right)$ in the plane of polarization for **ci** Pt/SiO_2_-SA and **cii** Pt/SiO_2_@TiO_2_ models simulated via FDTD method with the absorption maximum values of 415 and 515 nm, respectively. Reproduced with permission from Ref. [39]. Copyright © 2016 Springer Nature. Contrast of the measured **di** UV–Vis–NIR diffuse reflectance spectra (black) compared with the AM1.5 standard solar spectrum (orange) and **dii** photocatalytic hydrogen evolution performance for three core–shell structure models (SiO_2_/Pt/TiO_2_, SiO_2_/TiO_2_/i-Pt and SiO_2_/TiO_2_/Pt constructions) and blank groups. **e** Schematic illustration of two-electron transfer flows for photocatalytic hydrogen evolution over SiO_2_/TiO_2_/Pt construction. Reproduced with permission from Ref. [47]. Copyright © 2019 Wiley–VCH

The apparent light absorption induced by LSPRs could be reflected as a distinct resonance absorption peak in UV–Vis DRS measurement. Furthermore, the dynamic description of near field, consisting of the electromagnetic wave in interaction with quasi-free charges existing in plasmonic metals, is based on the solution of the Maxwell equation (a set of four partial differential equations). Various effective methods have been proposed to solve the Maxwell equation set in terms of LSPRs effect [37–40]. Mie theory explains the scattering of electromagnetic waves from the pure mathematical and physical perspective, which relies on the exact analytical solution of the Maxwell equation set [41, 42]. The scattered near-field could be divided into an infinite series of spherical multipole fields that are orthogonal at a sphere surface according to the Mie theory framework. It is worth noting that Mie theory is only targeted for homogeneous nanoparticles with spherical shape and is practicable for all possible ratios of the particle radius to wavelength. FDTD method regarded as a numerical solution to the Maxwell equation set is formulated based on the substitution of finite-difference terms for the spatial and temporal derivatives (Fig. 3ai) [43]. With the assistance of effective and efficient boundary procession, e.g., perfectly matched layer (PML) used for absorbing boundary condition (Fig. 3aii) [38], FDTD method has been employed for the light scattering model in terms of arbitrary shapes with variable size parameters [40, 44]. By Fourier transform, the frequency solution could be obtained by FDTD technique to deduce light transmission and reflection [45].

Considering that LSPRs adjustment has been widely studied from metal element (Au, Ag, etc.), shape (sphere, rod, array, etc.), and size (nanocluster, nanoparticle, nanolayer, etc.) aspects, tuning the dielectric environment of nanostructures has been recently proposed without the modification of plasmonic metallic nanostructures [46]. A unique light absorption model for the modulation of near-field dielectric scattering has been demonstrated by a designed self-assembled core–shell structure (labeled as Pt/SiO_2_@TiO_2_) with the controlled surface distribution of Pt nanoparticles (< 10 nm) [39, 47]. In this self-assembled Pt/SiO_2_@TiO_2_ construction, Pt nanoparticles are loaded on SiO_2_ spheres as the interior support, which are fine-coated with a thin TiO_2_ layer. For the scattering and absorption description of this unique structure, a coarse-grained multi-layer sphere model (Fig. 3bi) is computed by Mie theory method and a decorated core–shell model with explicitly embedded Pt nanoparticles inside the shell layer (Fig. 3bii) is otherwise computed by FDTD method [39]. The near-field intensity mappings derived from FDTD method as vividly depicted in Fig. 3d, Pt/SiO_2_@TiO_2_ structure exhibits a larger absorption cross-section and a more distinct absorption feature, in comparison with Pt/SiO_2_-SA (similar modal structure with Pt/SiO_2_@TiO_2_ except for the different refractive index of the encapsulating dielectric shell). This could be attributed to the obvious shift of near-field intensity toward Pt NPs’ vicinity by locating Pt NPs closer to the field maxima at a given scattering resonance. The intensified absorption coefficients of Pt NPs could be attributed to their localized absorption of scattered light in the near field of dielectric SiO_2_ surface, which is further facilitated by outer thin-layer TiO_2_ coating. The absorption descriptions for the near-field scattering model from Mie theory and FDTD computing have been verified by experimental measurements in terms of optical absorption ability and photocatalytic performance. Similar enhancements in the absorption of visible and near-infrared (Vis–NIR) light ranges are found for SiO_2_/TiO_2_/Pt and SiO_2_/Pt/TiO_2_ constructions in Fig. 3di, which could be attributed to the localized optical absorption of the scattered photons in the near-field of dielectric SiO_2_ surface from outmost Pt nanoparticles instead of conventional surface plasmon resonance from Pt. On the contrary, a weak enhancement in the absorption peak of Vis–NIR light ranges for SiO_2_/TiO_2_/i-Pt construction implies the imperfectly uniform spatial distribution of Pt loading by impregnation, which indicates the significance of regulated uniform distribution and suitable size of Pt NPs loading for the designed absorption model. In terms of photocatalytic hydrogen evolution (Fig. 3dii), the improved performances of SiO_2_/TiO_2_/Pt sample compared to blank samples could be ascribed to the additional injection of hot electrons by recycling scattered Vis–NIR photons in the near-field and transfer flow of excited electrons by Schottky junction from TiO_2_ semiconductor to Pt NPs, as illustrated in Fig. 3e. The key function of this new absorption model is elucidated as an additional utilization of the enhanced near field of resonant scattering mode by subtle metal nanoparticles. The absorption model applied on the tailored core–shell structure [48] as well as conventional LSPR effects, which could be verified by experimental results and theoretical simulations, would open a new avenue for the light absorption manipulation. By the absorption of scattered photons, the injection of energetic hot electrons that trigger photoredox reactions is beneficial to achieve higher photocurrents and better photocatalytic activity.

## Dynamics in Carrier Transfer and Separation

Under the irradiation, a massive injection of non-equilibrium carriers into a single-particle semiconductor photocatalyst with a micro-nano size is resulted from the interaction with incident photons. Energetic electrons are excited to the conduction band level whereas the rest electrons remain in the valence band level. In the period between the initial excitation and ultimate migration to reaction sites, photo-induced carrier pairs should get through separation and recombination processes. The separation behavior predominantly has been elucidated by Mott–Wannier mode for bulk semiconductors and Frenkel mode for organic molecules and polymers, where excitons are used to describe the bound states of photo-induced carrier pairs of which holes represent the collective behavior of confined unexcited electrons [49]. Generally, the smoothing separation could be modulated merely as exciton binding energy should be lower than 0.01 and 1 eV, and in turn, the binding energy is fundamentally associated with the effective mass and dielectric constant [4, 49]. Following separation, carrier pair diffusions could be impulsed by the intrinsic concentration gradient and the applied potential gradient, and therefore turn to the directional migration. The average time termed as carrier lifetime ($\tau$) during the diffusion migration is described by diffusion coefficient ($D$) and diffusion length ($L$). Herein, $D$ could be determined by Einstein Relation (Eq. 5) at a given drift mobility ($\mu$) and successively $L$ could be obtained (Eq. 6).

5 $$ D = \mu \frac{{{\rm K}_{B} T}}{q} $$

6 $$ L = \left( {D\tau } \right)^{1/2} $$

**Fig. 4:**
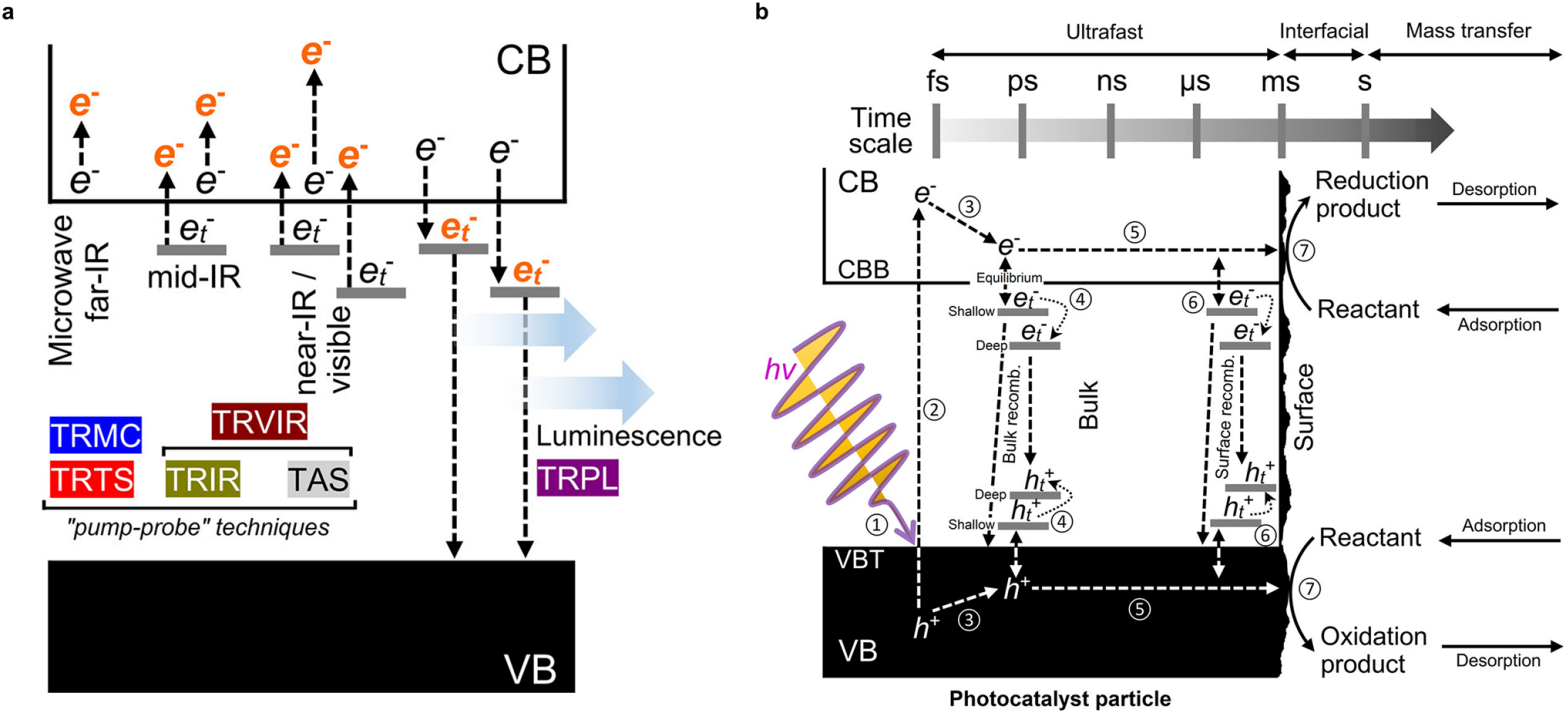
Schematics of various dynamic mechanisms detected with pump-probe techniques. **a** Illustration of different photo-induced electronic transition routes probed by various time-resolved spectroscopy measurements. **b** Illustration of the primary photophysicochemical processes involving photo-generated charge carriers with the corresponding time scales. Reproduced with permission from Ref. [59]. Copyright © 2023 American Chemical Society

It could be concluded that the mismatching of photo-induced carriers between the separation/migration rate and surface reaction rate leads to a higher bulk recombination rate. Besides, another mismatching reaction rate between electrons and holes is introduced as electrons are consumed more rapidly in the reduction reaction than holes in the oxidation reaction. Then, the excessive holes with a tendency to be trapped in surface sites are bound to form a potential drop unpinning the band edge resulting in severe surface recombination. For the improvement of the carrier dynamic transfer, the creation and enhancement of a built-in electric-field have been widely used, which is tightly associated with internal symmetry breaking and band structure engineering [2, 4, 50–52]. Various strategies have been proposed to introduce the built-in electric-field, including junction construction, morphological selective-facets control, asymmetric co-catalyst assembling and consecutive band bending [12, 13, 51, 53–55]. These strategies have been demonstrated for the promoted efficiency of carrier separation and transfer. Carrier lifetime is summarized and regarded as a significant parameter to describe the dynamic behavior of photo-excited charge carriers in separation and transfer processes within the bulk region of particulate photocatalyst nanomaterials, which could be adopted to assess the separation efficiency by apparent quantum efficiency value. In this review, we conclude the primary and advanced characterization techniques applied for the description of dynamic carrier behavior in photocatalysis. From the temporal perspective, transient spectroscopies have been widely applied to elucidate decay kinetics by photoluminescence and absorption signals from detected samples by pulse beam excitation (Fig. 4) [49, 56–58]. Besides, electrical signals also reflect charge carrier separation efficiency and migration paths in detected samples with applied bias potential in photoelectrochemical cells. With the cooperation of spectroscopies and microscopic imaging techniques, transfer behaviors of photo-induced carriers could be tracked and vividly displayed in the spatial resolution.

### Transient Absorption and Diffuse Reflectance Spectra (TAS and TDR)

**Fig. 5:**
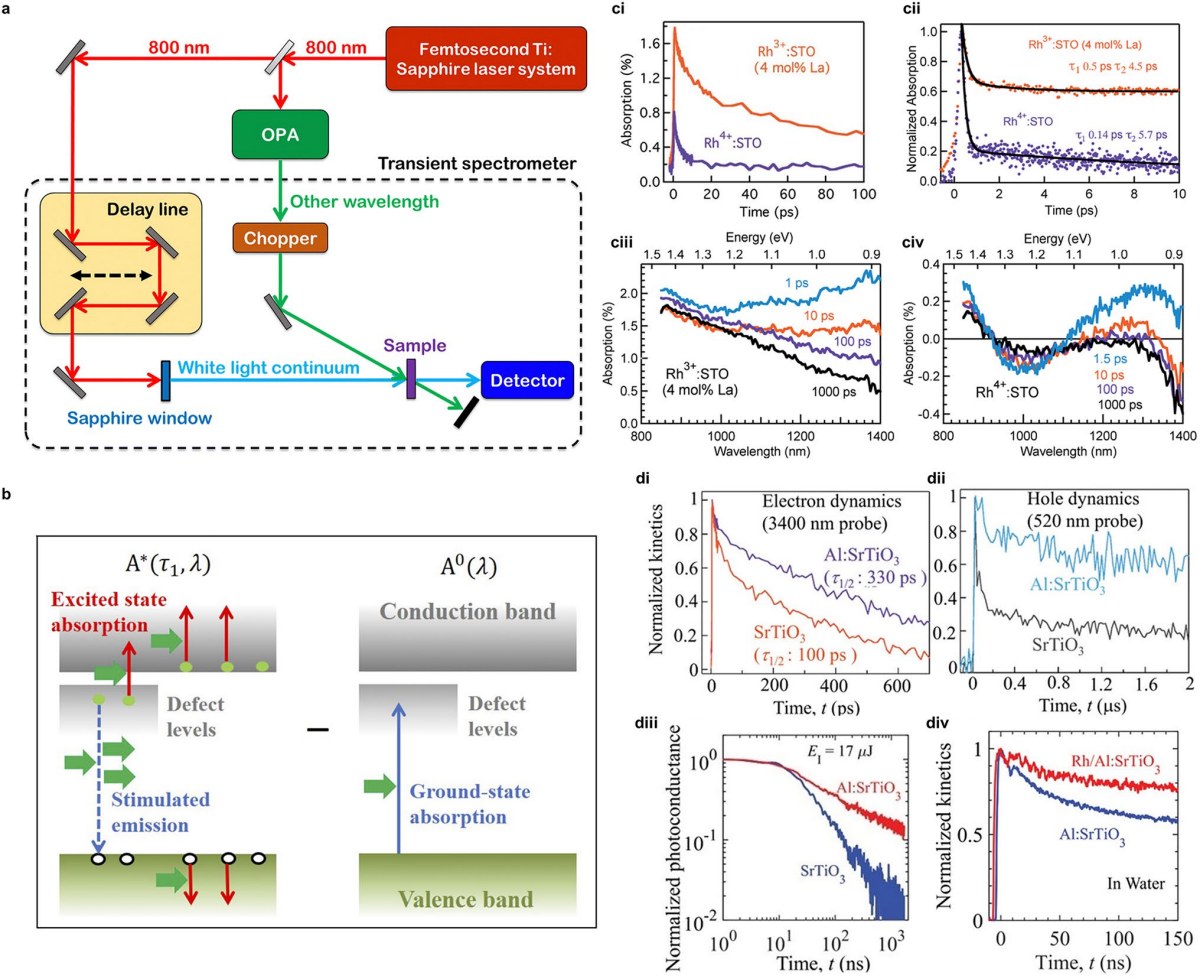
**a** Illustration of experimental setup for fs-TAS spectra detection and measurement. Reproduced with permission from Ref. [58]. Copyright © 2022 Royal Society of Chemistry. **b** Schematics of TAS signals originating from ESA, SE, and GSB routes. Reproduced with permission from Ref. [60]. Copyright © 2020 AIP Publishing. The comparisons between Rh^3+^:SrTiO_3_ and Rh^4+^:SrTiO_3_ samples in terms of **ci** fs-TDR decay (probe: 3435 nm), **cii** fs-TDR decay (probe: 920 nm) and **ciii-civ** TAS signals ranging from 850 to 1400 nm at different delay times with 400 nm pump. Reproduced with permission from Ref. [67]. Copyright © 2019 Royal Society of Chemistry. The comparison between pristine SrTiO_3_ and Al:SrTiO_3_ samples in terms of **di** fs-TDR signals (probe: 3400 nm, pump: 355 nm), **dii** μs-TDR signals (probe: 520 nm) and **diii** TRMC signals (detection: 8.753 GHz), which are characteristic for mobile/shallowly trapped electron dynamics, shallowly trapped/mobile hole dynamics and mobile electron dynamics, respectively. **div** Decay kinetics of normalized TDR signals before and after Rh deposition for Al:SrTiO_3_ (probe: 520 nm). Reproduced with permission from Ref. [69]. Copyright © 2023 Wiley–VCH

Transient absorption spectra (TAS) are developed on the basis of a pump-probe spectroscopic technique to measure the absorption energy of photo-induced excited states associated with carrier lifetime dynamics. As a pump-probe measurement, excitation of the sample is induced by a pump laser and the change in absorbance ($\Delta A_{{{\text{abs}}}}$) of the excited sample struck by a delayed probe laser is detected by transmission as a function of wavelength and time, in which transfer dynamics of electron and energy could be elucidated (Fig. 5a). The photons from a monochromatic light (pump source) excite electrons to leap from ground state to excited states and the photons from a broadband white light source (probe source) allow absorbance changes at the ground and excited states of sample to be measured as ($\Delta A_{{{\text{abs}}}}$). As shown in Eq. 7, $\Delta A_{{{\text{abs}}}}$ is contributed by excited-state absorption (ESA) as positive signals which contain photo-induced absorption (PIA) in the photochemical processes, ground-state bleaching (GSB) and stimulated emission (SE) as negative signals (Fig. 5b). Herein, ESA occurs in further excitation of excited electrons to higher excited level whereas GSB refers to electron depletion from excited states to ground states. Followed by fluorescence emission, SE represents the dropping from the first excited state minimum to vibrational energy levels on the ground, which displays a Stokes shift and is overlapped compared to GSB. Different from the above three photophysical processes, PIA known as product absorption is derived from photochemical processes with specific absorption changes caused by intermediate reaction species. Apart from classical electronic contributions, the thermal effect has been recently emphasized regarding to $\Delta A_{{{\text{abs}}}}$. By adjustment of optical delay device and electronic switch, a delay between pump light and probe light is modulated in the range of femtosecond to nanosecond. After the determination of pump light and probe light incident on the sample with perfect coordination in time and space, $\Delta A_{{{\text{abs}}}}$ of the sample with and without pump light excitation are accurately recorded in variable delay points. In the cases that SE and GSB are assumed as little contribution to TAS signals, the identification of photo-induced carriers at different states including free and trapped in/near band position is within reach. The addition of chemical hole (electron) scavengers and applied negative (positive) bias potentials has been innovatively used to detect clear and intensive electron (hole) signals in TAS.

7 $$ \Delta A_{{{\text{abs}}}} = \Delta A_{{{\text{ESA}}}} - \Delta A_{{{\text{GSB}}}} - \Delta A_{{{\text{SE}}}} $$

#### Dynamic Description

The transient absorption decay curve provides useful information in terms of the decay process types with corresponding dynamic rates and evidence for intermediate unstable electronic states, trap and surface states. There are three most commonly used fitting models to describe kinetic TAS decay curves as indicated below [56, 60]. (1) Multi-exponential decay (Eq. 8) contains several first-order kinetics terms expressed in a single-exponential formula and implies that each independent exciton as a single entity presents a simple decay depending on concentration only. The most prominent feature of first-order kinetic decay is the independence of lifetime on initial concentration. The first-order kinetics could be expected generally in the case that geminate pair recombination is the dominant route in carrier consumption. It should be noted that all terms of first-order kinetics in fitting equations must correspond to the identified substantial behaviors in carrier dynamics where additionally each behavior is supposed to be independent, and hence, the term amount is suggested as two or three usually. The carrier kinetics in maghemite derived from TAS is rationally described as a triple-exponential decay in picosecond time scale, which is further demonstrated as independent on excitation intensity. (2) Stretched-exponential decay is regarded as equivalent to a linear combination of single-exponential formulas in addition to the corresponding contribution of lifetimes (Eq. 9). In consideration of quantifying every characteristic lifetime, this fitting model could be expected to describe multiple simultaneous recombination routes that all present the single-exponential decay tendency, among which the dependent interrelationship is reflected by factor ($\beta$). In the case of ($\beta = 1$), the stretched-exponential model is identical to the multi-exponential model. (3) Power-law kinetic decay (Eq. 10) has a high correlation with carrier concentration and excitation intensity compared to the above exponential-based models. This model has been verified as a suitable fitting tool when it comes to limited carrier diffusion by primary trap-detrapping recombination. In some special cases, it is rather difficult to distinguish the power-law from the stretched-exponential kinetic model so that model combination is of necessity.

8 $$ \Delta A_{\tau } = \mathop \sum \limits_{i} \alpha_{i} e^{{ - \tau /\tau_{i} }} $$

9 $$ \Delta A_{\tau } = \mathop \sum \limits_{i} \alpha_{i} e^{{ - (\tau /\tau_{i} )^{{\beta_{i} }} }} $$

10 $$ \Delta {\text{A}}_{\tau } = \alpha \tau^{\beta } $$

#### Application

As a typical pump–probe technique, transient absorption spectroscopies could be classified by probing beam ranges as general TAS (Fig. 4a) for visible to near-infrared (IR) region, time-resolved IR spectra (TRIR) for mid-IR region and time-resolved visible to mid-IR spectra (TRVIR) for both two ranges simultaneously. Note that main characteristic absorption bands or peaks in ranges of < 4000, 5000–14000, and 15,000–25,000 cm^−1^ could be assigned to free or shallowly trapped electrons, deeply trapped electrons and trapped holes, respectively [59]. Therefore, TRIR with a lower probing energy should be more sensitive for free carriers and trapped carriers at shallow states while TAS is mainly targeted for trapped carriers at deep states. With the help of TRVIR, the facilitation or inhibition of photo-induced carriers to overall photocatalytic activities could be determined by simultaneously tracking the dynamic behavior of most carriers in kinetic decay analysis combined with their distributions and energy depth of trap states [61, 62]. Particularly for opaque samples with much stronger absorption ability, transient diffuse reflectance (TDR) is therefore updated by equipping TAS with reflectance collector and detector followed by Kubelka–Munk conversion [63, 64]. Apart from the above-mentioned transient absorption spectroscopies, time-resolved terahertz spectroscopy (TRTS) for far-IR region and time-resolved microwave conductivity (TRMC) for microwave region as two similar pump-probe techniques are designed for monitoring mobile photo-induced electrons in CB and holes in VB. Although TRMC has a limitation of attaching the sample to the non-conductive substrate in measurement and lacks a separate discussion of majority and minority carriers, it could convey direct messages in nanoseconds of the actual photoconductivity [65]. TRTS provides a contact-free approach to measure the photoconductivity in the sub-ps time scale [66].

The carrier dynamics in bulk SrTiO_3_ codoped with Rh and La to explain the effect of La doping level and electron transfer kinetics to reflect the effect of Rh doping states and Ru co-catalyst have been elaborately and comprehensively discussed via femtosecond TDR and TAS spectra [67]. The dynamic behaviors of photo-induced free electrons in CB and deeply trapped electrons are obtained by fs TDR profiles probed at 3435 and 920 nm, respectively (Fig. 5ci, cii). The low relevance between free electron kinetic decay and pump beam energy implies trapping is the major decay route rather than second-order direct pair recombination. The shorter half-lifetime $\tau_{1/2}$ of free electrons in Rh^4+^:SrTiO_3_ indicates that electron trapping occurs at in-gap energetically deep impurity Rh^4+^ level while deep trapping at oxygen vacancies could be expected due to the prolonged $\tau_{1/2}$ in Rh^3+^:SrTiO_3_. It is further demonstrated by facts that the unique signal rise around 100 ps in TDR profiles with 400 nm pump source is only observed by probing at 920 nm and the reduction in this signal rise is displayed by the substitution of 500 nm pump source. The absence of decay ranging from 850 to 1100 nm implies a more significant effect on electron trapping from the Rh^4+^ levels compared to oxygen vacancies (Fig. 5ciii, civ). In the comparison of free electron decay curves with and without Ru co-catalyst, electron transfer to Ru is beneficial for pair separation but is kinetically impeded by deep trapping at impurity level Rh^4+^. The states of dopants and the coupling of co-catalyst, which could be determined by TAS and TDR results, are supposed be selected after the elaborate consideration from multi-angles to realize the unhampered carrier separation and electron extraction from bulk to surface respectively, leading to the enhanced photocatalytic performance.

Leading-edge research about carrier dynamics in La,Rh codoped SrTiO_3_ photocatalyst sheet half electrodes has been reported by potential-dependent TAS measurements [68]. Spectroelectrochemical results of La,Rh:SrTiO_3_ indicate that the positive TA signals at 1250 nm which is assigned to CB electrons are fitted with the power-law kinetic model, which keeps unaltered over the range of applied potentials, while on the contrary, two kinetic decay regimes are regulated by the applied potential for Rh:SrTiO_3_. A distinct transient bleach with loss of absorption at 1250 nm, which could be attributed the loss of Rh^4+^ states (primary electron trapping states), is found at positive potentials and positive signals with similar power-law decay to La,Rh:SrTiO_3_ reappear with the shift toward negative applied potentials. As a result of the co-doping effect of Rh and La on SrTiO_3_ characterized by TAS, the roles of dopant states and minority carrier lifetime accompanied by electronic structure reconstruction and Fermi level effects as primary factors should be paid more attention to develop advanced photocatalyst devices. Similar effects of Al doping with Rh doping on carrier dynamics in SrTiO_3_ have been investigated by the combination of TDR and TRMC [69]. With Al doping to SrTiO_3_, shallowly trapped & mobile electrons and holes both exhibit a slower decay trend with longer lifetimes derived from fs TDR probed at 3400 nm and μs TDR probed at 520 nm, respectively (Fig. 5di, dii). From photoconductivity decay features in TRMC (Fig. 5diii), carrier pair recombination routes are determined as band-to-band recombination by decay rates in early-time (< 100 ns) and recombination of mobile electrons with detrapped holes by that in late-time (10^–1^ to 1 μs). A reduction of n-type behavior introduced by Al doping contributes to a prolonged carrier lifetime (from 50 ns to 12.5 μs), increased diffusion length (from 883 nm to 14 μm) and decreased recombination rate (from 2 × 10^7^ to 8 × 10^4^ s^−1^), which is concluded from numerical calculation calibrated with parameters obtained by TDR and TRMC. The slower kinetics of ns-hole decay (Fig. 5div) is found after Rh deposition because of the extraction of electrons and the reduction of bimolecular recombination derived from Rh co-catalyst. The suitable modulation of doping level by aliovalent dopant states is recommended to be used for the suppression of adverse reduced or oxidated state as the unexpected traps for the severe carrier recombination and consequently better photocatalytic water splitting performance, as demonstrated by comprehensive TAS, TDR and relevant pump-probe spectral measurements.

Due to the fact that carrier transfer routes of GSB and SE could be deduced from the steady-state UV–Vis absorbance and steady-state photoluminescence measurements, TAS has been widely coupled with other spectroscopies to comprehensively describe photo-induced carrier dynamics. The similar tendency of carrier kinetic decay for SrTiO_3_ is derived by TAS and TRPL spectra [70], which could be described as a slow decay fitted by a single exponential with tens of nanoseconds under weak excitation followed by a fast decay fitted by nonexponential within subnanoseconds under high excitation. The good consistency in decay times τi obtained by TAS and TRPL signals is resulted from that same rate equation could be used for calculation, which implies that intrinsic Auger recombination dominates photo-induced carrier decay dynamics under high excitation.

### Time-Resolved Photoluminescence Spectra (TRPL)

**Fig. 6:**
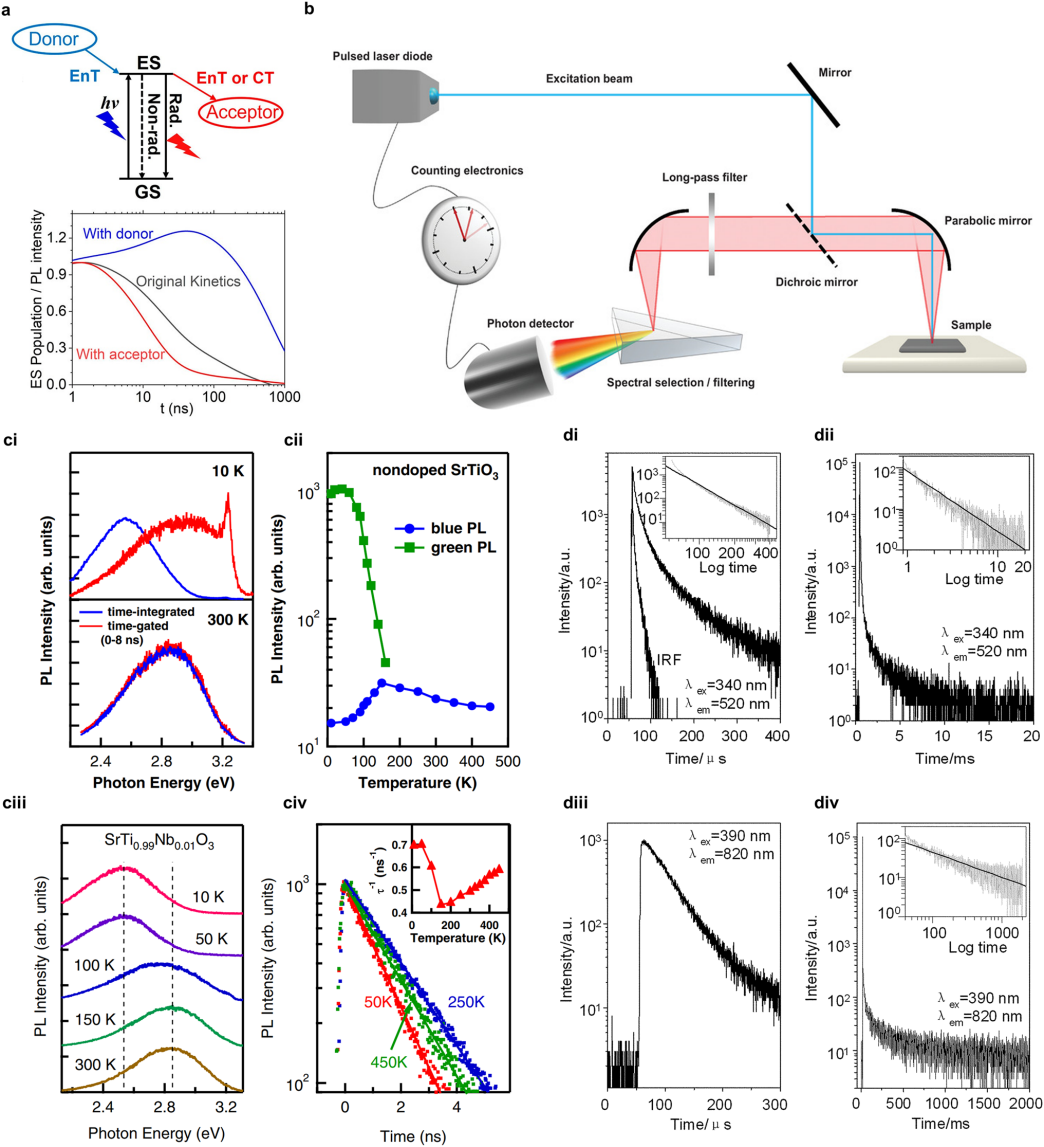
**a** Illustration of PL signal generation and doping effect on PL decay kinetics. Reproduced with permission from Ref. [73]. Copyright © 2022, AIP Publishing. **b** Experimental setup of TRPL spectra detection and measurement. Reproduced with permission from Ref. [77]. Copyright 2020 Wiley–VCH. **ci** Time-gated and time-integrated PL spectra, **cii** temperature dependence of PL intensities in terms of the broad blue and green PL bands for nondoped SrTiO_3_. **ciii** PL spectra and **civ** TRPL decay curves of doped SrTiO_3_ under different temperatures. Reproduced with permission from Ref. [80]. Copyright © 2009 American Physical Society. **di–dii** TRPL decay curves of anatase and **diii–div** rutile TiO_2_ with the inset of log–log plots. Reproduced with permission from Ref. [81]. Copyright © 2010 Royal Society of Chemistry

Photoluminescence (PL), which is regarded as an indication of the re-emitted light, originates from the radioactive decay of electrons populated at the photo-excited states. In a typical measurement, a polarization followed by semiconductor Bloch equations is initially generated by excitation light at a certain wavelength (photon energy larger than band gap) of which photo-induced electrons and holes have finite momenta in the valence band and conduction band, respectively. Photons are emitted due to the recombination where electrons and holes undergo energy and momentum relaxation, which consequently are detected as photoluminescence spectra [71]. Basically, excited carrier populations and transmission paths could be provided by steady-state photoluminescence spectra from peak intensity and emission wavelength, respectively. For further temporal behaviors, time-resolved photoluminescence spectra are a useful tool to probe the emission profile that decays with time after a pulsed laser beam (Fig. 6b), which provides insights into the dynamics of carrier lifetime [72–74]. The time resolution varies from femtoseconds to nanoseconds equipped with fluorescence detectors, such as optical gating and time-correlated single-photon counting techniques [75–77].

#### Dynamic Description

The recombination behavior could be elucidated in two dynamic descriptions. As the recombination occurs once the carrier lifetime is shorter than the required migration time, it could be divided into first-order (Shockley–Read–Hall recombination, $\kappa_{1}$, second-order (band-to-band recombination, $\kappa_{2}$) and third-order (Argur recombination, $\kappa_{3}$) according to the kinetic rates (Eq. 11) [49]. The kinetics of the bulk recombination is variable in different photocatalytic materials, which depends on defect concentration in lattice integrity, crystal morphology with size effect, and electronic configuration of band structure. From the thermodynamic aspect, it is inevitable that electrons tend to fall back to the valence band, which subsequently eliminates holes via the annihilation and releases energy by electronic relaxation. Therefore, according to energy release types, the recombination behavior mainly contains the radiative type with photon emission and the non-radiative type with phonon vibration (Fig. 6a). Band-to-band recombination that primarily occurs in direct band gap semiconductors have both the radiative and non-radiative types, whereas defect-induced Shockley–Read–Hall recombination from trapping at impurity energy level and Argur recombination belong to the non-radiative type [73, 78]. Generally, the fast component of PL decay pattern usually in multiexponential fitting in nanosecond and picosecond scales is resulted from band-edge emission. The fastest component is induced by non-radiative Auger recombination and trapping.

11 $$ \frac{{{\text{d}}n}}{{{\text{d}}\tau }} = - \kappa_{1} n - \kappa_{2} n^{2} - \kappa_{3} n^{3} $$

#### Application

Pioneering femtosecond dynamics of band-edge emission for CdS nanocrystals (NCs) is conducted by TRPL characterization [79]. The discrete PL features indicate the presence of shallow localized states in the band gap and the corresponding emission explains the extremely fast buildup dynamics in 400–700 fs range. The PL decay of a short lifetime band is derived from the fast capture of photo-induced holes with ps scale while the decay of emission is caused by slower trapping of electrons with 20–30 ps lifetime. Due to its higher sensitivity at a single NC level, simplicity and availability, TRPL is proven effective for the detection of carrier multiplication (CM) and efficiency measurement and further demonstrates the generation of multiple excitons within a single NC after absorption of a single photon from both dynamical and spectral perspectives. The observation of CM and biexcitons are realized by dynamical and spectral signatures from PL patterns, respectively. There is the same spectral shift in the early-time PL between two excitation conditions with high-energy photons at low intensity and low-energy photons at high intensity. The ultrafast extraction of multiple carriers from NCs following CM instructs us to apply CM effect for the significant improvement of apparent quantum efficiency in photocatalytic systems with the collaboration of NCs or QDs and classic photocatalyst nanomaterials [16].

As for typical semiconductor photocatalyst SrTiO_3_, the carrier dynamics have been revealed by TRPL over a range of temperatures [70, 80]. The intrinsic and extrinsic carrier recombination could be reflected by a board blue PL band (~ 3.2 eV) in pristine SrTiO_3_ and a green PL band (~ 2.5 eV) in electron-doped SrTiO_3_. For blue PL in the pristine sample, the decay profile is composed of a nonexponential fast decay within subnanoseconds and a single-exponential slow decay within several nanoseconds, which is perfectly fitted by Eq. 11 as the second-order part is negligible for SrTiO_3_ (Fig. 6c). The former one occurs in the low-density region attributed to non-radiative single-carrier trapping, whereas the latter one ascribed to non-radiative Auger recombination located at the high-density region. In contrast, the dynamics in the electron-doped sample are dominated by Auger recombination between the doped carrier and photo-induced carrier of which the decay curve is single-exponential given by Eq. 12. According to the comparison of temperature, phonon-assisted Auger recombination is predominated in temperature-dependent dynamics and a high carrier mobility contributes to a high carrier trapping rate in the near band-edge state of blue PL at a lower temperature. The carrier behaviors in terms of carrier mobility and carrier lifetime elucidated by TRPL offer a prediction to distinguish the dependence of variable defect-related modifications on the resultant photocatalytic performance, which might be positive for extension or negative for prevention.

12 $$ \tau_{1/e}^{ - 1} = \left( {\kappa_{1} + \kappa_{2} n_{0} + \kappa_{3} n_{0}^{2} } \right) $$

It has been reported that carrier lifetime is prolonged (milliseconds) under weak excitation with microseconds duration, which indicates the tight link between recombination processes and trap states. In the excitation condition of the pulsed laser beam with a higher intensity, trap-filling effect accelerates the recombination rate within nanosecond scale [73, 74]. Therefore, trap concentration and trap depth with the determination of various combination types in different phases of TiO_2_ have been described via TRPL equipped with a mircosecond flash lamp at a cryogenic temperature [81, 82]. The carrier pair recombination rate is considered as highly correlated with photo-induced carrier migration. For anatase phase of TiO_2_, dynamics of carrier pair recombination display a power-law decay tendency, which is ascribed to the trapping-detrapping effect. The two regions from fast (400 μs) and slow (20 ms) time-ranges in the decay curve are assigned to the direct and indirect trapping processes, respectively (Fig. 6di, dii). In contrast, a power-law component and an exponential-law component are observed in the recombination dynamics of rutile phase. The recombination paths could be elucidated as the photo-induced carrier pair being captured at stable trap states and the relaxation via trap-to-trap hopping successively. In anatase TiO_2_, the fast decay could be attributed to carriers trapping at oxygen vacancies while the slow decay could be assigned to the carriers quenching from shallow defect states to luminescent sites via trap-to-trap hopping, which eventually emits visible luminescence. In rutile TiO_2_, one part of the contribution to near-infrared luminescence produced by the direct trapping path is similar to anatase TiO_2_ (Fig. 6diii, div). As the other part, indirect trapping path includes quenching to conduction band minimum and to deep non-radiative energy level, which are both followed by relaxation to luminescent sites. Thus, a relatively high photocatalytic performance of anatase might be ascribed to the participation of trapped carriers with slow decay in comparison to rutile which produces considerable deep defect trap states. Concluded from the contrast of TRPL spectra between anatase and rutile TiO_2_, the slower decay kinetics of photo-excited carrier behavior for anatase than that for rutile consequently results in the enhanced photocatalytic hydrogen evolution, which could be ascribed to the difference of defect types. It could be elucidated as the transfer behavior of photo-excited electrons trapped in oxygen vacancies in anatase is found much better than that of electrons trapped in intrinsic deep defects in rutile, which highlights the regulation of defect-related engineering by defect type, defect location and defect density [82].

### Surface Photovoltage Spectra (SPV)

Surface photovoltage spectroscopy has emerged as an advanced surface characterization technique equipped with an optical pump-probe method to study and monitor the separation and transfer behavior in space of photo-generated charge carriers. Due to the fact that the injection of photo-generated charge carriers in responsive to external light irradiation is bound to induce the carrier transfer, the resultant spatial redistribution of carrier pairs leads to the increase/decrease in contact potential difference of sample surface which is recorded as surface photovoltage (SPV) [83]. In the classical semiconductor interface (e.g., p-type), the flowing or trapping of majority charge carriers (positive holes) at the surface causes the non-uniform distribution of carrier population and induces the charge space region so called as the depletion layer with the reduced concentration of majority charge carriers. In the charge space region, an intrinsic built-in electric-field pointing from surface side to field free region in bulk side is hence established, a downward band bending is constructed and a potential barrier for holes (a potential well for electrons) is formed [84]. Non-equilibrium charge carrier pairs generated by photo excitation are driven by the surface electric-field for separation, which contributes to the reduced degree of band bending. The consequent change in surface potential is probed as a negative SPV signal. The surface photovoltage with high sensitivity ranging from nV to mV can reflect sequential carrier separation and transfer processes including diffusion, drift, polarization, trapping, etc. As simulated, SPV signal is highly correlated with (nearly proportional to) the multiplication involving the total amount of photo-generated charge carriers in separation and the transfer distance (Eq. 20) [85]. Furthermore, SPV techniques have been extended to time-resolved and spatial-resolved characterizations with the assistance of a fixed planar capacitor and a Kelvin-probe (KP) loaded on atomic force microscopy (AFM), respectively.

#### Dynamic Description

For the cases where carrier transport is mainly dominated by diffusion instead of drift by electric-field, SPV could be derived from the separation behavior of carrier pairs and then described as the double integration of Poisson Equation. These cases are appropriate with low carrier density, small sample size in nanoscales and surface potential induced by photon irradiation. Specifically, in SPV measurement with a fixed planar capacitor in terms of most conventional semiconductors, the separation process could be applied in the illustrated parallel plate capacitor configuration because the external fixed space charge region primarily contributes to the carrier separation distance [86]. A simplified assumption that the distance between positive and negative charge carrier centers could be described as the carrier separation distance. In a simple layer model with a certain amount $N\left( t \right)$ of positive charge carrier located at the outer-side surface and the same amount of electrons with a distribution $n\left( {x,t} \right)$ diffusing into the layer with a thickness $L$, SPV could be obtained by integration (Eq. 13) of the local electric-field intensity $E\left( {x,t} \right)$ , which is originated from the local charge density by Poisson equation (Eq. 14).

13 $$ {\text{SPV}} = \mathop \smallint \limits_{0}^{L} - E\left( {x,t} \right){\text{d}}x $$

14 $$ \frac{\partial E}{{\partial x}} = - \frac{ne}{{\varepsilon_{0} \varepsilon }} $$

SPV could be obtained by a second integration with the assumption of $E\left( {L,t} \right) = 0$ as follows (Eq. 15).

15 $$ {\text{SPV}} = \frac{e}{{\varepsilon_{0} \varepsilon }}\mathop \smallint \limits_{0}^{L} {\text{d}}x\mathop \smallint \limits_{0}^{x} n\left( {s,t} \right){\text{d}}s $$

Based on the planar capacity illustration, the local charge distribution that induces the local voltage (Eq. 16) could be transferred to a thin slice of differential charge $dq$ (Eq. 17). The photovoltage could be presented in an easy interpretation (Eq. 18). The center of negative charge carriers $\left[ {x_{n} } \right]\left( t \right)$ is expressed as the mean position of electrons (Eq. 19).

16 $$ d\left( {{\text{SPV}}} \right) = \frac{dq}{C} = \frac{xdq}{{\varepsilon_{0} \varepsilon S_{Area} }} $$

17 $$ \frac{{{\text{d}}q}}{{{\text{d}}x}} = e \cdot n\left( {x,t} \right) \cdot S_{{{\text{Area}}}} $$

18 $$ {\text{SPV}}\left( t \right) = \frac{e}{{\varepsilon_{0} \varepsilon }}\mathop \smallint \limits_{0}^{L} x \cdot n\left( {x,t} \right){\text{d}}x = \frac{e}{{\varepsilon_{0} \varepsilon }}N\left( t \right)\left[ {x_{n} } \right]\left( t \right) $$

19 $$ \left[ {x_{n} } \right]\left( t \right) = \frac{1}{N\left( t \right)}\mathop \smallint \limits_{0}^{L} x \cdot n\left( {x,t} \right){\text{d}}x $$

The deduction of SPV could be completed (Eq. 20) in the presence of both negative and positive charge carriers in the layer, where $\left[ {x_{p} } \right]\left( t \right)$ is the center of positive charge carriers. The carrier separation distance $d\left( t \right)$ is the difference between the negative center and the positive center.

20 $$ {\text{SPV}}\left( t \right) = \frac{e}{{\varepsilon_{0} \varepsilon }}N\left( t \right)\left( {\left[ {x_{n} } \right]\left( t \right) - \left[ {x_{p} } \right]\left( t \right)} \right) = \frac{e}{{\varepsilon_{0} \varepsilon }}N\left( t \right)d\left( t \right) $$

It could be concluded that both the total amount of charge carriers in separation $N\left( t \right)$ and carrier separation distance $d\left( t \right)$ make major contributions to SPV generation. This calculation could be speculated for general spatial distributions of negative and positive charge carriers in the bulk.

For the cases in which carrier separation is driven by the built-in electric field (e.g., space charge region), a simplified description for SPV could be proposed referring to the open-circuit potential (Eq. 21) [85]. $\Delta n$ is the injected concentration of non-equilibrium charge carriers by photo-excitation and $n_{0}$ is the minority charge carriers in thermal equilibrium.

21 $$ {\text{SPV}} = \frac{kT}{e}\ln \left( {1 + \frac{\Delta n}{{n_{0} }}} \right) $$

#### Application

The normal SPV measurements assisted with a Kelvin-probe are effective to directly record the induced change of contact potential difference before and after light irradiation, which is tightly associated with the performance of photo-generated carrier separation and relevant band structure parameters of band offsets and band gap. Besides, in SPV measurements assisted with a fixed planar capacitor, the measurement capacitor formed between the sample electrode and the reference electrode is measured and SPV signals denoted as the potential change of sample surface potential can be detected from the voltage change of the measurement capacitor in the external circuit based on a metal–insulator-semiconductor construction. The measurement capacitor is shunted by a measurement resistance which is introduced to set zero potential in the initial and a high-impedance buffer is applied to acquire the accurate voltage of measurement capacitor photovoltage close to sample photovoltage. Modulated SPV technique is developed to exclude irrelevant photo-induced processes resulting in the surface potential change, i.e., charge exchange by photothermal effect and carrier trapping at deep defect states. Transient SPV techniques can be realized using a fixed planar capacitor without a feedback loop to eliminate electrostatic forces, which is essential in SPV assisted with KP-AFM [87–90]. The fixed capacitor equipment provides high sensitivity in the orientation perpendicular to the plate and in the high temporal resolution. Modulated SPV technique is favorably applied for ultra-thin-layer structures and single-nanoparticle with an elaborated detection for the low carrier density in 10^8^ cm^−2^ scale and small space in 1 nm scale [91]. Transient SPV measurements have been selected to elucidate the dynamics of carrier separation in variable processes. However, the average information detected by conventional SPV techniques is obscure for complex photocatalytic systems, because multiple separation and transfer behaviors of photo-generated carriers are included. The combination of SPV measurements and imaging location techniques has been realized for achieving simultaneous temporal and spatial high-resolution in the dynamic description of carrier separation and transfer [92–95].

**Fig. 7:**
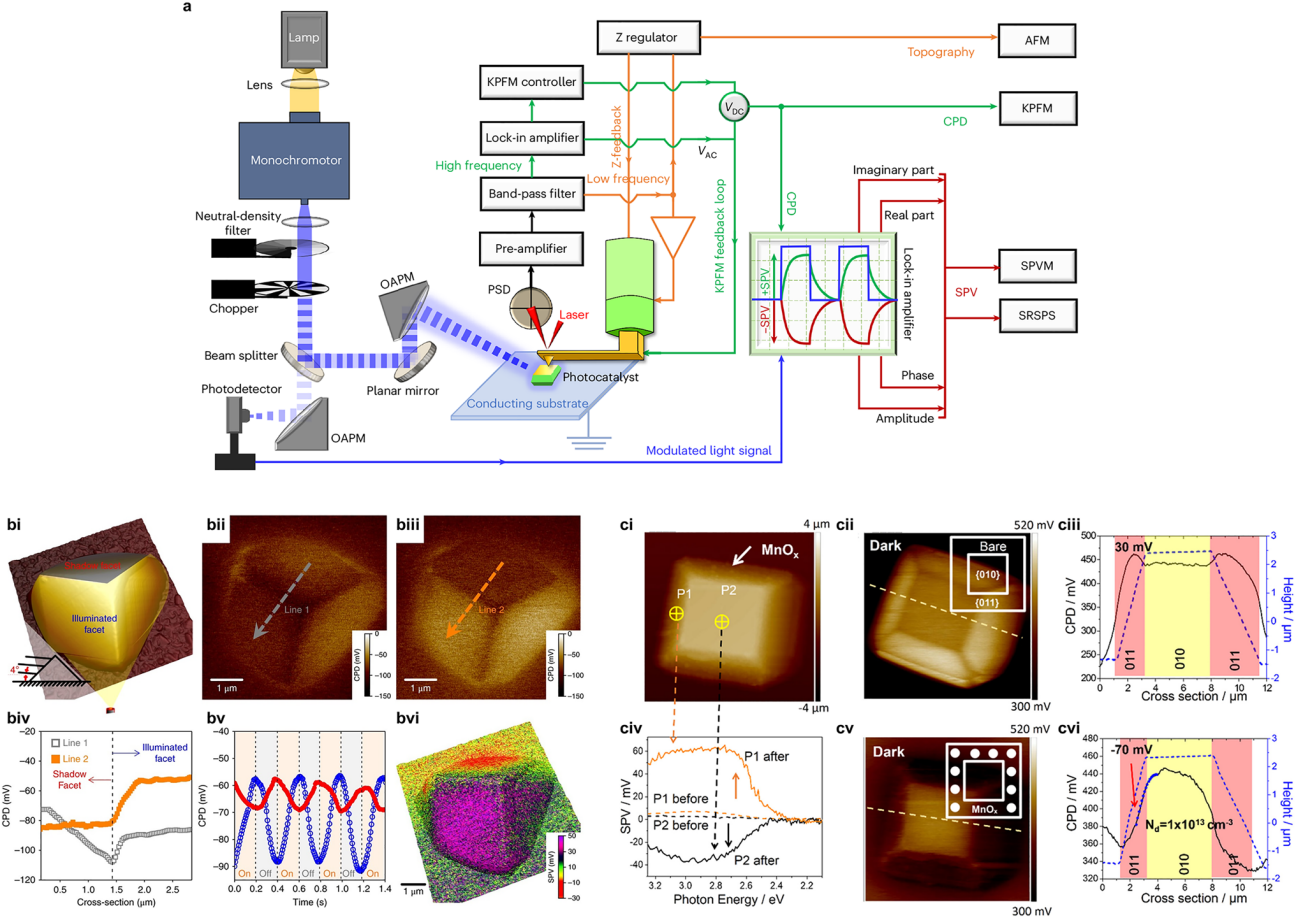
**a** Illustration of AFM-based microscopy and spectroscopy measurements with multiple functions. Reproduced with permission from [92]. Copyright © 2024, Springer Nature. **bi–bvi** Description for charge separation between the illuminated facet and shadow facet of a single Cu_2_O cube via KP-AFM assisted with SPV. **bi** Schematics of asymmetric irradiation experiment. **bii–biii** Surface potential profiles by KPFM under **bii** dark and **biii** illumination conditions with **biv** the corresponding line distribution. **bv** Transient signals derived from shadow (red) and illuminated (blue) facets and **bvi** SPVM imaging after subtraction. Reproduced with permission from Ref. [97]. Copyright © 2018 Springer Nature. **ci–cvi** Description for charge separation between anisotropic facets with asymmetric co-catalyst assembly. **civ** Contrast of SR-SPV signals measured from {011} (P1) and {010} (P2) facets of single BiVO_4_ crystal, labeled in **ci** AFM imaging, **cii** before and **civ** after MnO_x_ selective-deposition, respectively. The contrast of line distributions in terms of height and surface potential signals measured from KP-AFM imaging **ciii** before and **cvi** after MnO_x_ selective-deposition. Reproduced with permission from Ref. [98]. Copyright © 2017 American Chemical Society

SPV microscopy (SPVM) and spatial-resolved SPV (SR-SPV) with spatial resolution in the nanometer scale and electric-resolution in the sub-mV scale have been developed for visualizing surface potential influenced by photo-induced changes mapped on nanoscale imaging of photocatalyst particles [96–100]. Illumination systems loaded on KP-AFM (Fig. 7a) and transient SPV combined with microscopy techniques, such as scanning electron microscopy (SEM), scanning tunneling microscopy (STM) and photoemission electron microscopy (PEEM), are designed as powerful tools for SR-SPV measurements [101–104]. Conventional KP-AFM can output the surface height and contact potential difference (CPD), which offer sample information on particle morphology and surface potential mapping respectively. In SPV measurements assisted with KP-AFM (middle part in Fig. 7a), SPV signals are calculated as the difference of surface potentials in dark and light conditions, which could be quantitatively denoted by CPD signals. Illumination systems (left part in Fig. 7a) induce the photoexcitation to yield SPV signals and the modulation of SPV signals via light modulations in terms of frequency and intensity by chopper and neutral-density filter, respectively. In addition, SPV amplification and processing systems (right part in Fig. 7a) are introduced to increase the accuracy and sensitivity of modulated SPV signals, which are analyzed as amplitude signals and phase signals after being operated in the lock-in amplifier taking the synchronous signal as the reference signal converted from part of modulated light by a photodetector. In detail, modulated SPV signals by signal-amplitude modulation show less signal noise and higher sensitivity than conventional CPD signals which avoids the severe influence of tip-lift height. Besides, modulated SPV signals by phase analysis unravel the carrier separation direction and differentiate multiple dynamic mechanisms. Phase signals in ranges of 90° to 180° shifted from 180° and − 90° to 0° are ascribed to electron-dominated and hole-dominated carrier separation behaviors, respectively. Specifically, phase angles approaching 0° or 180° and a phase lag of 90° points to fast and low carrier separation and relaxation routes, respectively [92].

The photo-generated carrier separation controlled by intrinsic diffusion due to the different mobilities of electrons and holes is revealed for a high-symmetry Cu_2_O crystal under asymmetric light irradiation, by SPV mapping with nanometer resolution is collected by KP-AFM (Fig. 7b) [97]. Based on the localized SPV signals (Fig. 7biv) from the illuminated facet (increased by 30 mV) and the shadow facet (decreased by 10 mV), the opposite photo-induced surface potential changes indicate different carriers are generated by asymmetric photo excitation (Fig. 7bvi). Combined with periodic modulation of illumination between light-on and light-off (Fig. 7bv), photo-generated holes and electrons are inclined to be separated and transferred to the illuminated facet and the shadow facet, respectively. An additional SPV of 20 mV is provided by the carrier separation by diffusion through the migrations of holes and electrons toward the illuminated facet and the shadow face. It indicates that the diffused carrier separation is stronger than the drifted carrier separation by surface electric-field in SCR, which only yields a SPV of 10 mV. The dominant driving force by gradient diffusion originates from the huge difference (approximately 100 times) in the mobilities of holes and electrons as well-fitted simulations calculated with the photo-Dember effect. The carrier separation dynamics are further improved by spatially asymmetric assembly of co-catalysts controlled by the externally manipulated illumination, which results in the aligned built-in electric-field with a directional driving force of 90 mV. Similar phenomena are found from the SPVM observation for BiVO_4_ single crystal (Fig. 7c) [98]. The modulation of spatially loading dual co-catalysts (Pt and MnO_x_) leads to a significant enhancement of the local electric-field (~ 2.5 kV cm^−1^) with a vast increase in local SPV signal (~ 80 times), which elevates interfacial carrier transfer by aligning the vectors of surface built-in electric-field. The intensified built-in electric field visualized and quantified by SPVM measurements demonstrates the above-mentioned symmetric breaking strategies, asymmetric illumination, anisotropic facet exposure, asymmetric assembly of co-catalysts and variable junction engineering as effective modifications for the enhanced carrier separation and elevated photocatalytic efficiency.

Coupling the time-resolved technique (in femtosecond scale) and space-resolved PEEM (in micrometer scale) is considered effective in probing ultrafast SPV dynamics [93, 102, 105, 106]. The carrier dynamics within the space charge layer of MoS_2_ flakes have been comprehensively studied by a transient micro-area SPV measurement [106]. The relaxation of SPV signals as obtained from the decay of transient SPV provides carrier dynamic information in terms of the recombination behavior with the lifetime parameter. The increasing excitation intensity results in a larger SPV maximum and a shorter plateau followed by a faster decay, which could be ascribed to the acceleration of Shockley–Read–Hall recombination related to trapping effects. The population of carriers preferring to recombine is larger than that preferring to detrapping because of the almost fully filled trapping sites. The positive correlation of SPV signals and temperature reflects two mechanisms the higher temperature causes the larger band bending and causes more carriers to be detrapped from defect states. The systematic investigations of variable competing carrier behaviors, including trapping, detrapping and recombination by transient SPV cooperated with microscopy reflect the potential application of layer-structure 2D material design in terms of photocatalytic and optoelectronic devices. With regard to the carrier separation dominated by defect trapping and detrapping, transient SPV as a function of decay time and photon energy is an effective tool to unravel the dynamic behavior mechanism [90]. In the time scale less than 100 ns, the fast negative SPV signals irrelevant to surface defect are primarily influenced by fast charge separation due to the downward band bending in SCR. In the time scale of μs to ms, the decreased negative SPV signals under excitation beyond band gap are resulted from the carrier diffusion/recombination and the relaxation of SPV signals is guided by trapped carriers at intrinsic defect states that prolongs the lifetime of separated carrier pairs driven by built-in electric-field in SCR. The importance of near-surface defect regulation and defect-related engineering are verified for the promotion of photo-excited carrier separation and total photocatalytic energy-conversion efficiency by the combination of time- and space-resolved SPV techniques.

## Dynamics in Interfacial Reactions

Apart from the proportion of photo-induced carrier pair diminished by the recombination process, the rest arrives at the localized near-surface region through separation and migration and participates in the interfacial chemical reactions. On the solution side, the reactant molecular and ionic species transfer from the bulk solution in diffusion and get anchored at surface sites in adsorption. The surface sites turn out to be active sites where the high-energy electrons and holes carrying considerable reducing and oxidizing capacities respectively accumulate. Then the adsorbed species interact with these carrier pairs at different active catalytic sites where the product species are generated from interfacial redox reactions. The soluble product with the increased concentration is inclined to diffuse to bulk solution after desorption while on the contrary, the segregation of insoluble species is more complicated with additional kinetic restrictions [107, 108]. In the classical photocatalytic water splitting involving hydrogen evolution reaction (HER) and oxygen evolution reaction (OER), hydrogen and oxygen molecules are generated and distributed adjacent to the solid surface. With the accumulation to the saturation concentration, dynamic behaviors of the evolved gas bubbles including the initial nucleation, gradual growth and ultimate detachment and coalescence [109, 110]. However, the long-time scale of gas bubble behaviors ranges from ms to s is significantly unmatched with the time scale of the antecedent carrier behaviors in carrier excitation, separation and transfer (fs-μs) [9]. The slow dynamics in the gas bubble behavior increase the reverse reaction rate. Besides, the long-time occupation of surface-active sites by the undetached bubbles also would suppress the forward interfacial reactions, leading to the overaccumulation of carrier pairs near the surface and the increasing probability of recombination [2, 4, 111]. As the interfacial reactions would become the rate-determining step, especially for the photocatalytic involving gas evolution, it is necessary to decouple the dynamics in interfacial reactions. The spatial distribution and temporal evolution of reactant and product species at surface active sites are proposed and highly stressed as key parameters to describe the dynamic behavior of photo-excited charge carriers participating in the interfacial photocatalytic reactions on the surface region of particulate photocatalyst nanomaterials. The carrier behavior in the interfacial reactions could be reflected by the indicator molecule, intermediate and product species, and attached bubbles, which could be probed by direct microscopy observation and transient spectral measurements. Specifically, the behaviors of the indicator molecule and evolved bubbles could be described by fluorescence microscopy whereas the intermediate and product species could be traced by time-resolved infrared spectroscopy.

### Single-Molecule Fluorescence Imaging

**Fig. 8:**
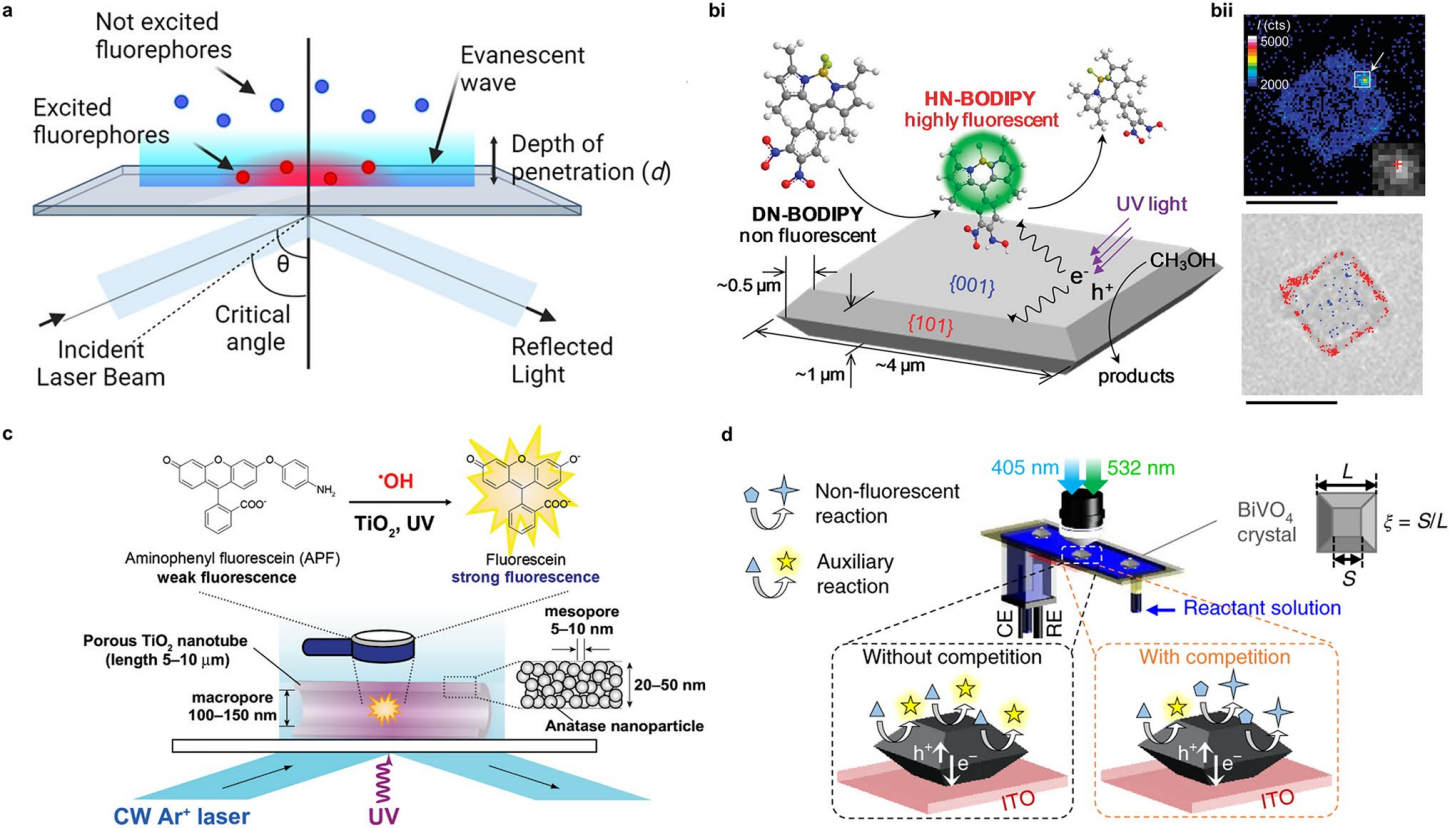
**a** Illustration of TIRFM measurement for single-molecule fluorescence imaging. Reproduced with permission from Ref. [120]. Copyright © 2023 by the authors. Licensee MDPI. **bii** Fluorescence mapping (up) via TIRFM with electron transmission imaging (down) of a single immobilized TiO_2_ crystal, which is probed by **bi** the photocatalytic generation of fluorescent HN-BODIPY from non-fluorescent DN-BODIPY. Reproduced with permission from Ref. [116]. Copyright © 2011 American Chemical Society. **c** Tracing visualization of molecular transport on a single TiO_2_ nanotube via TIRFM probed by the photocatalytic generation of fluorescein from aminophenyl fluorescein. Reproduced with permission from Ref. [119]. Copyright © 2009 American Chemical Society. **d** Schematic experimental setup of COMPEITS technique via a wide-field fluorescence microscopy under two-laser epifluorescence illumination. The photocatalytic reactions in terms of non-fluorescent target and fluorescent auxiliary reactions occurring on a single BiVO_4_ crystal in a photoelectrocatalytic microfluidic cell. Reproduced with permission from Ref. [125]. Copyright © 2019 Springer Nature

Single-molecule fluorescence microscopy (SMFM) is emerging as a super-resolution microscopy to study variable photophysical and photochemical processes. The fundamental mechanism of SMFM is to detect the characteristic fluorescence signal of the single target molecule that serves as a fluorogenic probe by chemical activations. In principle, the fluorogenic probes are those special molecules that initially are not responsive to fluorescence excitation and could be converted to the activated fluorescent state during the surface catalytic reaction [112–114]. Some sophisticated measurements assisted with advanced detection equipment are designed to meet the requirement of detecting weak fluorescent emission from a single target molecule, including total internal reflection fluorescence microscopy (TIRFM) and confocal fluorescence microscopy (CFM) [115–119]. Herein, TIRFM is widely applied for dynamic behaviors of immobilized interfacial reactions due to the relatively low background noise by the evanescent field (Fig. 8a), while CFM is often used for the diffusing molecule in solution [120]. In addition, the modified fluorescence microscopy measurements display promising advantages in probing the single-molecule reaction with high spatiotemporal resolution. Generally, the corresponding fluorescence spatial distribution could be visualized by collecting fluorescence signals represented for the location of surface reaction sites, with an approximate Gaussian profile and Airy pattern for intensity mapping [113]. Due to the superiority of a single target molecule, spatial imaging could reach the tens of nanometer level. The temporal resolution highly depends on the combined high-speed imaging cameras, e.g., electron-multiplying charge-coupled device (EM-CCD), complementary metal–oxide–semiconductor (CMOS), which could be improved to sub-millisecond and sub-nanosecond levels [112, 113, 120, 121]. Besides the advantage of the high spatiotemporal resolution, SMFM could be set in some in-situ environments as optical measurements are conducted under ambient and liquid solution conditions compared to other electron microscopy characterizations.

#### Dynamic Descriptions

The dynamic behaviors of electron transfer beneath surface and kinetic reactions of intermediates via real-time tracking fluorescence imaging sequence with quantitative and statistical analysis of individual fluorescence behavior. The on-times and off-times for the fluorescence bursts noted as ($\tau_{{{\text{on}}}}$) and ($\tau_{{{\text{off}}}}$), respectively, are used for the description of fluorescence life. Despite these stochastic parameters, the inverse terms ($\tau_{{{\text{on}}}}^{ - 1}$) and ($\tau_{{{\text{off}}}}^{ - 1}$) reflect the rate constants for the production, adsorption and desorption reactions of the probe molecule [113, 114, 122]. For the stabilized interfacial reactions in the condition that the adsorption rate of the probe molecule is much faster than the activation rate, Langmuir–Hinshelwood model is recommended for processing SMFM results [115, 122, 123]. The turnover frequency ($\upsilon_{{{\text{TOF}}}}$) could be obtained by the effective activation rate of the probe molecule ($\upsilon_{e}$), the equilibrium constant of the probe molecule adsorbed onto the surface ($K_{{{\text{ad}}}}$) and the concentration of the probe molecule ($c$) as follows (Eq. 22). Most common calculations are converted by the inverse item ($\tau_{{{\text{off}}}}^{ - 1}$) and the number of fluorescence bursts in fluorescence imaging. In the case that another inverse item ($\tau_{{{\text{on}}}}^{ - 1}$) is correlated with the concentration ($c$), the correlation indicates the desorption behavior of the probe molecule from the surface. In contrast, the non-correlation implies the probability of self-detachment.

22 $$ \upsilon_{\text{TOF}} = \tau_{\text{off}}^{ - 1} = \frac{\upsilon_{e} \times K_{\text{ad}} \times c}{1 + K_{\text{ad}} \times c} $$

#### Application

A series of fluorescence spectroscopy and microscopy measurements probed by appropriate indicator molecules have been conducted to describe dynamic behaviors of charge carriers at surface and elucidate kinetic mechanisms of interfacial reactions, based on single-particle models in photocatalytic and photoelectrocatalytic systems. The anisotropic transfer flow of photo-generated carrier pairs between {001} and {101} facets of TiO_2_ single-particle is vividly reflected by facet-dependent fluorescence mapping (Fig. 8b) [116]. The indicator probe molecule (DN-BODIPY) is developed for the fluorogenic reaction where the substrate (DN-BODIPY) is converted to the fluorescent product (HN-BODIPY) based on the photo-induced intramolecular ET mechanism [117]. The in situ single-particle spatial observations for fluorescence location distribution are achieved by TIRFM, and in the meanwhile, the kinetic single-molecule temporal detections for fluorescence lifetime distribution are realized by CFM coupled with a time-correlated single-photon counting system (TCSPC). The formal fluorescence location distribution is shown by the density of fluorescence bursts with higher intensities than background signals after centroid analysis. The frequency of observed fluorescence bursts represents the fluorogenic reaction rate. The obvious difference rate estimated as 25 and 102 counts μm^−2^ min^−1^ (*c* = 2 μM) for {001} and {101} facets directly demonstrates the facet-dependent transfer behavior that photo-generated electrons preferentially flow to {001} facets. The latter fluorescence lifetime is presented in a Gaussian distribution after biexponential fittings and the shorter lifetime (~ 2.6 ns) than than in bulk solution (~ 3.7 ns) implies the possible interfacial electron migration from fluorescent molecules to TiO_2_ photocatalyst. The facet-dependent preference of photo-excited carrier separation, demonstrated by in situ observation of the interfacial photocatalytic reaction via TIRF, encourages the development of heterogeneous facet-selective photocatalysts by facet engineering strategy. Besides, a unique heterogeneous spatial distribution of photocatalytic sites is found in the individual TiO_2_ porous nanotube by TIRFM. The probe surface reaction is the conversion from aminophenyl fluorescein (APF) to fluorescein with the participation of hydroxyl radicals (^•^OH), which is generated by photocatalysis on TiO_2_ nanotube [119]. The transport behavior of reagents within the nanotube structure is clearly visualized by the spatiotemporal observations, which is found highly associated with interfacial photocatalytic reactions (Fig. 8c). Revealed by spatiotemporal emissive fluorescein mapping results, the efficient reagent transport accelerates the photocatalytic activity, which enlighten us to design the spatial heterogeneity such as porous-structure nanomaterials. Similar to SMFM based on fluorescence detection, spatial-resolved photoluminescence spectroscopy (SRPL) is originated from inherent PL signals in photocatalysis as discussed above without additive fluorescent probe molecules. A combination of a single-particle photoluminescence spectroscopy and a single-molecule spectroelectrochemistry spectroscopy is developed to investigate the transfer behavior and reaction dynamics of photo-generated carriers for individual TiO_2_ nanowire [122, 124]. The intrinsic effect of bulk and surface defect states on carrier transport is uncovered by temporal PL measurements. The quenching kinetics of defect-trapped electrons is further probed by the introduced interfacial reaction with molecular O_2_ and is described as a Langmuir–Hinshelwood model. The relationship between the heterogeneous distribution of defect sites and the location distribution of PL active sites supports the existence of long-distance carrier transport along the nanowire. By the observation of SRPL results, the efficiency of carrier separation is further improved by the long-distance carrier transport, which implies the better photocatalytic performance by the introduction of defects with a heterogeneous distribution.

For the interfacial reactions without any fluorescent species involved, an innovative concept of competition-enabled imaging technique with high-resolution (COMPEITS) has recently been proposed [125]. The non-fluorescent reaction process could be equivalently observed due to the suppression or enhancement of fluorescence intensity and the shift of emission wavelength for the auxiliary fluorescent reaction process so-called as competition. The fluoregenic conversion from AR molecules to fluorescent product Resorufin is detected as the auxiliary reaction by SMFM with two-laser epifluorescence illumination (Fig. 8d), to probe the non-fluorescent surface photoelectrocatalytic oxidation of HQ to Quinone by single-particle photocatalyst (truncated BiVO_4_). The effects of size and shape parameters from variable crystal-facet exposure on the kinetic parameters of non-fluorescent surface photodegradation are fully revealed by the quantitative spatial observation in super-optical resolution (nanometer level) in the sub-particle level. The adsorption efficacy of reactant species (HQ) resulted from edge effects is found as biphasic dependences on the shape parameter in an intermediate size range. Furthermore, the inter-facet junction effects in terms of photocatalytic surface reactions are comprehensively studied by multimodal functional imaging measurements [126]. It assembles the collaborative designs of photoelectrochemical current mapping in the sub-facet level, super-high-resolution spatial imaging for the carrier-involved reaction by wide-field epifluorescence illumination, and the whole single-particle photoelectrochemical current measurement. The selective distribution of photo-excited carrier pairs for anisotropic facets of the single nanoparticle could be mapped in high solution by SMFM, which indicates that asymmetric facet-junction engineering is demonstrated as a useful strategy for more effective spatial separation of carriers.

### Time-Resolved Infrared Spectra (TRIR)

**Fig. 9:**
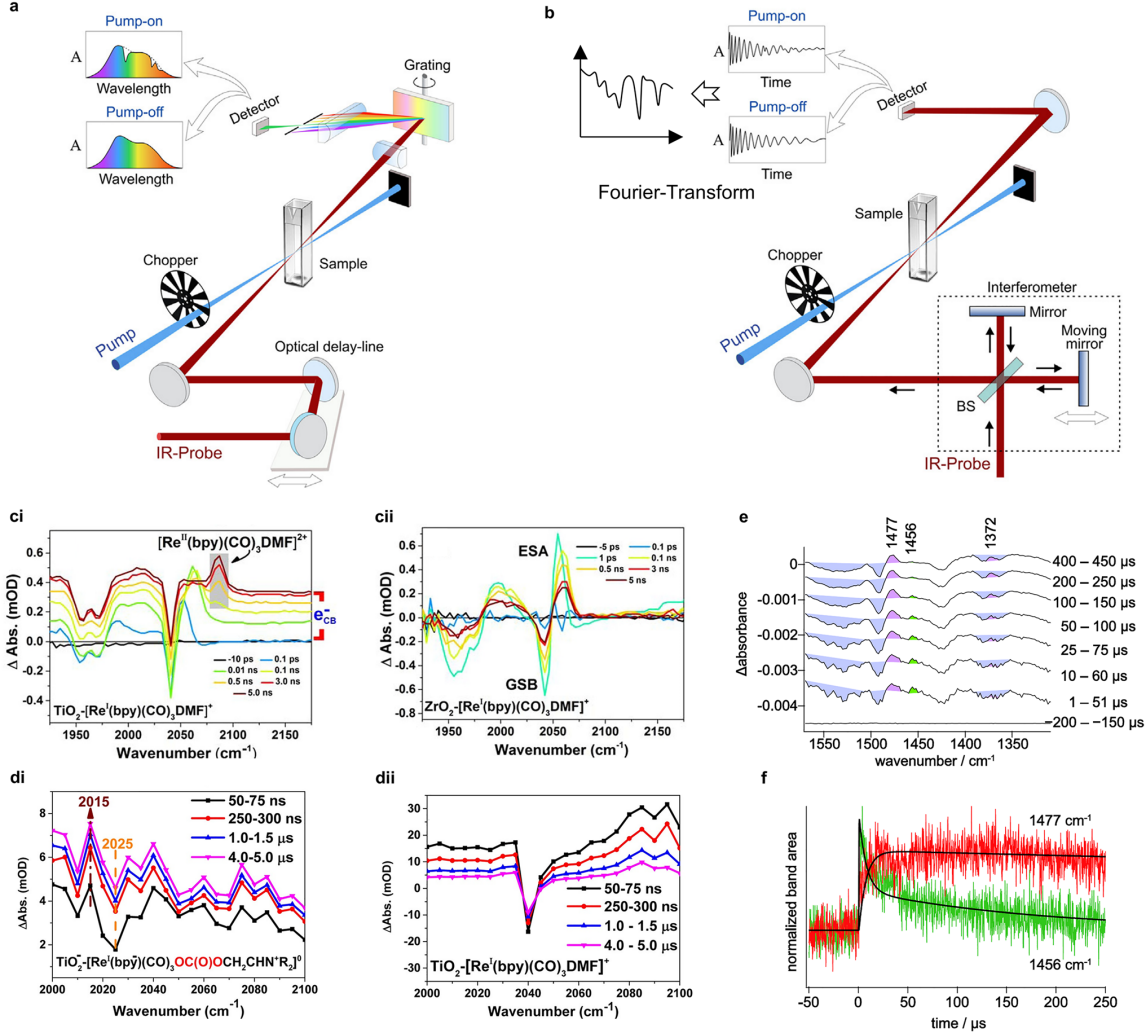
Illustrations of TRIR measurement with dispersive spectrometer-based setup (**a**) and FTIR measurement interferometer-based setup and Fourier transformation of this interferogram (**b**). Reproduced with permission from Ref. [127]. Copyright © 2020 Elsevier. The comparison of fs-TRIR detected from TiO_2_–[Re^I^(bpy)(CO)_3_]^+^ system (**ci**) and ZrO_2_–[Re^I^(bpy)(CO)_3_]^+^ system in DMF solution without TEOA at CO_2_ atmosphere (**cii**). The collection of ns-TRIR at variable time delays detected from TiO_2_–[Re^I^(bpy)(CO)_3_] system (**di**) and TiO_2_–[Re^I^(bpy)(CO)_3_DMF]^+^ system (**dii**) in the presence of TEOA/CO_2_ after photoexcitation. Reproduced with permission from Ref. [129]. Copyright © 2016 American Chemical Society. **e** Collection of FTIR at variable time delays for the photocatalytic system of Pt/TiO_2_ in exposure to pivalic acid gas. **f** The corresponding temporal profiles of FTIR peak intensities for the transient bands at 1477 cm^−1^ (product) and at 1456 cm^−1^ (transient species) with fitting lines. Reproduced with permission from Ref. [133]. Copyright © 2013 Elsevier

Time-resolved infrared spectroscopy as a transient pump–probe technique has similar measurement mechanisms and experimental setups compared to the above-mentioned TAS characterization. In the fundamental dispersive spectrometer-based setup for TRIR detection (Fig. 9a), an IR detector receives the probe beam through a spectrometer where the broad range can be dispersed by a grating device [127, 128]. Pump beam is regulated by a chopper and ultimately filtered after the arrival of the sample. However, here it should be noted that for TRIR, the probe equipped with mercury cadmium telluride (MCT) detectors ranges in the mid-IR wavelength section compared to TAS with probe wavelengths ranging from the visible to near-IR region by silicon-based detectors. Due to the lower probing energy, TRIR has the advantage of targeting at the dynamic behavior of charge carriers in the free transport and shallow trapped sites, while TAS mainly focuses on carriers in deep trapped sites [59]. TRIR with mid-IR region probing signals avoids the mutual interference of multiple carrier behaviors, because the signals contributed by GSB process associated with band gap and SE process derived from photoluminescence are located in UV–Vis and near-IR regions. Hence, TRIR with distinguishable signals is predominating in direct tracing for dynamic intermediate reactions connected with photo-generated carriers and molecular intermediates. Fourier transform (FT) IR spectrometer has been developed to tackle the deficiency of IR detectors in terms of low sensitivity and slow risetime, which is referred to the interferometer-based IR setup for FTIR detection (Fig. 9b). Two replicas are generated through an interference, reflected back by two individual mirrors and recombined to incident onto sample. The accuracy of the difference in optical paths of two replicas could be reached as one mirror can be adjusted and the other is fixed, which is regarded as a conventional rapid-scan FTIR. The spectral resolution of rapid-scan FTIR method depends on the difference in the length of optical path scanned for the interferogram, which is higher than the dispersive spectrometer limited by grating efficiency. Hence, a high production of IR spectra in a short time could be achieved by rapid-scan FTIR. In contrast, a step-scan mode has been proposed to realize the time-resolved FTIR as it can deal with one main problem of rapid-scan mode as explained. There is an inevitable mutual interference between the time-difference from the response of sample being pumped and time-difference from spectral multiplexing during the interferogram acquisition.

The functions of synthetic molecular catalysts introduced for the enhancement in the photocatalytic performance could be elucidated by the investigation of the excited states associated with the electron injection, which could be reflected by TRIR measurements. For typical phosphonated molecular catalyst [Re^I^Br(bpy)(CO)_3_]^0^, the photocatalytic mechanism of photoreduction from CO_2_ to CO is revealed by detecting CO stretching vibrations, which is regarded as a pointer to the back-donation from Re to CO, in the region from fs to s via TRIR [129]. In TRIR comparison (Fig. 9ci–cii) of the molecular catalyst attached to inert ZrO_2_ and active TiO_2_, a new peak observed in the larger wavelength region and a wide absorption band are resulted from the oxidized state of the molecular catalyst and the electron injection to TiO_2_ CB, respectively. In the presence of electron donor TEOA, merely TRIR signal for the electron injection is observed with the disappearance of the signal for the excited state, which indicates the oxidized catalyst would be reduced by TEOA oxidation. With the assistance of ns-laser for a long-time scale detection (Fig. 9di–dii), a new peak observed in lower wavelength region which increases vastly within the first 100 ns implies the increased electron density of central Re ion with a single-electron reduced state. In the same time scale, the simultaneous increase in broad absorption band signal for injected electrons at TiO_2_ CB supports that a singly reduced catalyst is formed by accepting the electron from the oxidized TEOA radical. Therefore, the electron injection triggers the sequential two-electron oxidation of TEOA with the first and second electrons releasing in ps and μs time scales, respectively. The decay kinetics confirms that the electron injection is rapid in time scale from ps to ns while the lifetime of injected electrons at TiO_2_ CB is in time scale from ms to s. The overall photocatalytic enhancement is attributed to the high oxidation ability and slow recombination rate of the oxidized Re species and the considerable reservoir capacity of TiO_2_ for electron storage and release. As TRIR can provide additional kinetic and structural information and monitor vibrational energy flows and dissipations, it has been used for investigating ultrafast photochemical electron transfer (ET) processes [130]. In the study of photo-induced ET involving Re^I^(CO)_3_(N,N) moiety, TRIR helps to explain the faster ET rate than the theoretical value. The excitation of pristine state derived from vibration and solvation causes the ultrafast interligand electron transfer (ILET) of [Re(4-N-methylpyridinium-pyridine) (CO)_3_(N,N)]^2+^ case in ps time scale. The case of tryptophan oxidation by excited rheniumchromophores exhibits the accelerated rate of ET from excited-state Trp to ^*^Re^II^. The inverse ET of rhenium-porphyrin assembly demonstrates the acceleration effect of the produced hot ground state on a highly exergonic process in the Marcus inverted region. The accelerated photo-induced ET process demonstrated by TRIR helps to elucidate the better efficiency of solar energy conversion in artificial photosynthesis systems on a molecular level.

The generation and consumption of reactive intermediates and active species could be precisely and timely captured by TRIR, which is described as the dynamic footprint for interfacial reactions. In terms of water oxidation, two intermediates are identified as the surface superoxide for three-electrons oxidation of which the absorption signal is detected at 1013 cm^−1^, and oxo Co(IV) site for one-electron oxidation of which the absorption peak emerging at 840 cm^−1^ by FT-TRIR [131]. The FTIR in rapid scanning in the attenuated total reflection (ATR) mode records the temporal dynamics of two intermediates, which shows different catalytic sites responsible for the generation of two intermediates. In situ FTIR combined with TRIR spectroscopies as effective tools monitor the active sites and surface species in the photocatalytic reactions of methanol on Pt/TiO_2_ photocatalyst [132]. The characteristic TRIR decay of photo-excited long-lived electrons has a good correlation with hydrogen evolution rate. It reveals that long-lived electrons with a kinetic decay in ms to s timescales contribute to the hydrogen reaction route ($2e_{tr}^{ - } \left[ {{\text{Pt}}} \right] + 2{\text{H}}^{ + } \to 2{\text{H}}\, \cdot \, \to {\text{H}}_{2}$). The function of loaded Pt can be concluded as the adsorption of molecularly methanol or water and mediation of proton transfer on TiO_2_ surface, which highlights the significance of metal co-catalysts loaded on particulate nanomaterials for the improved photocatalytic performance. TRIR in the time scale from μs to ms is applied to analyze photodegradation of pivalic acid by Pt/TiO_2_ [133]. From the spectral contrast between steady-state and time-resolved IR measurements, the emergency of negative bands and peaks at 100–150 μs after photo excitation implies the launch of photodegradation (Fig. 9e). The negative bands are identified as pivalate attached to the catalyst and gaseous pivalic acid. The primary transient species corresponding to the negative peaks at 1456 cm^−1^ are demonstrated as t-butyl radical and the product is ascribed to isobutane (at 1477 cm^−1^) (Fig. 9f). The consistency between the decay dynamic behavior of the intermediate with 7.3 μs lifetime and that of the product supports the conversion route from t-butyl radical to isobutane. The discovery of main product as isobutane instead of isobutene by FTIR helps to determine the reaction of pivalate on the surface of rutile TiO_2_ single-crystal, which offers designing ideas for the reaction route in photocatalytic systems. The TRIR signals for C=O stretching mode are detected as two absorption bands corresponding to the final product (acetone, at 1700 cm^−1^) and the intermediate species (anion radical of acetone, at 1640 cm^−1^) for the solution oxidation of 2-propanol by Pt/P25 photocatalyst [134]. The mechanism analysis of photocatalytic reaction kinetics revealed by TRIR provides proof of effective modifications for photocatalyst materials.

## Conclusion and Perspective

The comprehensive description for dynamic behaviors of photo-induced charge carriers in photocatalytic systems has received increasing attention in recent years, in consideration of the undesired kinetic mismatching among the consecutive tandem steps in mass and energy flows. The dynamic descriptions, including the simulation of photon flux distribution in the irradiation field, the reflection and evaluation of charge carriers in the separation and transfer, and the probing and tracing detections in the interfacial reactions, have been realized via variable advanced characterizations and techniques from the spatiotemporal aspects. In the pioneering research works and breakthrough findings, the magnitudes of temporal and spatial resolutions have been reached fs scale and nm scale, respectively, which benefits us in figuring out the kinetic bottleneck and improving the rate-limiting process correspondingly. In summary, three primary steps in photocatalytic systems (regarded as optical absorption step, carrier separation and transfer step and interfacial reaction step) are still encountered with respective dynamic obstacles and bottlenecks in each step, which require specific determination methods and characterization measurements to solve the targeted problem and study the principal contradiction in the key process. For the optical absorption issue, the irradiation field distribution involving crucial parameters, e.g., LVRPA, could be simulated by RTE solution and FDTD method. For the carrier transfer issue, the separation and recombination behaviors of photo-excited charge carriers described by pivotal parameters, e.g., carrier lifetime and decay kinetics, could be obtained by pump-probe techniques (including TAS, TDR, TRPL, SPVM) endowed with high-resolution temporal and spatial analytic functions. For the interfacial reaction process, the surface carrier behavior occurring in photocatalytic redox reactions is reflected by the special probing molecules, which could be tracked by fluorescence indication measurement (e.g., SMFM technique). Besides, the spatial distribution and temporal evolution of reactant and product species at surface active sites could be further monitored by transient FTIR measurements. Nevertheless, the reported characterizations for dynamic descriptions in photocatalytic processes still have restrictions and challenges as summarized.

- In terms of the description for light absorption and irradiation field, it is relatively hard to conduct experimental measurements and real-time detected mapping for the quantitative analysis of radiation distribution, compared to the elaborate numerical simulations assisted by full and accurate calculation and modeling methods. The modulations of photon flux paths and radiation distribution rely on the medium and photocatalyst, which makes it complicated to design and modify the photoreactor systems.
- In terms of the description for separation and transfer behavior of charge carriers, operando characterization measurements are highly emphasized and significantly required with more realistic information. It should be noted that the difference between the measurement conditions in different atmospheres, with/without medium and sacrificial agents, would introduce an interference in the assessment of the carrier separation efficiency.
- Besides, the trajectory and destiny of charge carriers are very complex and hard to distinguish among various behaviors. Therefore, it is suggested to apply variable complementary techniques to accomplish the collaborative measurements for multidimensional descriptions, including multiple spectral and microscopic techniques. Despite the developed coupling techniques, the resolution capability would be sacrificed, which means ultrahigh temporal and spatial scales are uneasy to be obtained simultaneously in the current technology.
- For the probing of interfacial reactions, the constraint of probing species and targeted reactions and the requirement of a high-quality single-particle nanomaterial render it challenging to extend and generalize for variable cases. The universality and applicability of the target interfacial reactions should be taken into consideration.
- The development of an integrated relation or a unit parameter is imperative to provide the synthetical consideration and evaluation for photocatalytic systems so that we could establish a direct and clarified relationship between the designed key factors and the consequent overall performance. It would facilitate us to construct and design photocatalyst nanomaterials following the principle that introduction and improvement of the key parameters are supposed to be given priority, which might simplify the selection and construction of targeted semiconductor nanomaterials as particulate photocatalysts.
